# Increased Hemodynamic Load in Early Embryonic Stages Alters Myofibril and Mitochondrial Organization in the Myocardium

**DOI:** 10.3389/fphys.2017.00631

**Published:** 2017-08-30

**Authors:** Madeline Midgett, Claudia S. López, Larry David, Alina Maloyan, Sandra Rugonyi

**Affiliations:** ^1^Biomedical Engineering, Oregon Health & Science University Portland, OR, United States; ^2^Multiscale Microscopy Core, OHSU Center for Spatial Systems Biomedicine, Oregon Health & Science University Portland, OR, United States; ^3^Proteomics Core, Oregon Health & Science University Portland, OR, United States; ^4^Knight Cardiovascular Institute, Oregon Health & Science University Portland, OR, United States

**Keywords:** cardiac development, hemodynamically-induced cardiac remodeling, outflow tract development, congenital heart disease, embryonic myocardial maturation, hemodynamic regulation of heart development

## Abstract

Normal blood flow is essential for proper heart formation during embryonic development, as abnormal hemodynamic load (blood pressure and shear stress) results in cardiac defects seen in congenital heart disease (CHD). However, the detrimental remodeling processes that relate altered blood flow to cardiac malformation and defects remain unclear. Heart development is a finely orchestrated process with rapid transformations that occur at the tissue, cell, and subcellular levels. Myocardial cells play an essential role in cardiac tissue maturation by aligning in the direction of stretch and increasing the number of contractile units as hemodynamic load increases throughout development. This study elucidates the early effects of altered blood flow on myofibril and mitochondrial configuration in the outflow tract myocardium *in vivo*. Outflow tract banding was used to increase hemodynamic load in the chicken embryo heart between Hamburger and Hamilton stages 18 and 24 (~24 h during tubular heart stages). 3D focused ion beam scanning electron microscopy analysis determined that increased hemodynamic load induced changes in the developing myocardium, characterized by thicker myofibril bundles that were more disbursed in circumferential orientation, and mitochondria that organized in large clusters around the nucleus. Proteomic mass-spectrometry analysis quantified altered protein composition after banding that is consistent with altered myofibril thin filament assembly and function, and mitochondrial maintenance and organization. Additionally, pathway analysis of the proteomics data identified possible activation of signaling pathways in response to banding, including the renin-angiotensin system (RAS). Imaging and proteomic data combined indicate that myofibril and mitochondrial arrangement in early embryonic stages is a critical developmental process that when disturbed by altered blood flow may contribute to cardiac malformation and defects.

## Introduction

The heart is the first functional organ in the embryo, and starts beating by the coordinated interaction of primitive myofibril bundles and energy supplying mitochondria as soon as the early heart tube is formed (Wainrach and Sotelo, 1961). These premature organelles are not regularly organized as in the mature heart. Instead, disarrayed myofibril-like structures and mitochondria are distributed throughout the cytoplasm and lack alignment and packing in early embryonic myocytes (Smolich, 1995). Throughout normal development, myofibril and mitochondrial cell volume fractions increase, myofibril bundles align to the longitudinal axis of the myocyte (which themselves align in the direction of maximal contraction), and myofibrils together with mitochondria arrange in an orderly pattern (Fischman, 1967; Manasek, 1970; Brook et al., 1983; Rai et al., 2008). These developmental changes take place in order to ensure optimal force transfer and contraction in the heart (Raeker et al., 2014). Mature myofibrils emerge in late embryonic stages, and are characterized by an aligned and organized repeated pattern of thick (actin) and thin (myosin) filaments that are connected by sarcoplasmic-derived Z-discs (Manasek, 1970). Further, in late fetal and adult periods, myocardial mitochondria are stacked in orderly rows within a myofibrillar lattice, and contact surrounding ATP consumption sites to aid in contraction (Roberts et al., 1979; Vendelin et al., 2005).

Blood flow dynamics play a critical role in regulating early cardiac morphogenesis (Culver and Dickinson, 2010). Hemodynamic forces exerted on cardiac tissue walls trigger mechanotransduction mechanisms that lead to physical, chemical, and gene regulatory responses (Davies, 1995). Numerous studies have shown that surgically altered blood flow results in a spectrum of cardiac defects seen in human congenital heart disease (CHD; Clark and Rosenquist, 1978; Clark et al., 1989; Hogers et al., 1997, 1999; Sedmera et al., 1999; Tobita et al., 2002; Hu N. et al., 2009; Midgett and Rugonyi, 2014), however the ways in which altered blood flow triggers malformation remain unclear. Understanding the root causes of abnormal development in embryonic stages that leads to congenital heart defects is essential for future prevention and treatment.

This study specifically investigated myocardial changes in myofibril and mitochondrial organization following altered hemodynamics in the chicken embryo cardiac outflow tract. The outflow tract connects the primitive ventricle with the arterial vessel system in the early embryonic tubular heart. Outflow tract remodeling is frequently studied because the outflow tract gives rise to structures that are often involved in congenital heart defects including the aorta, pulmonary trunk, a portion of the interventricular septum, and semilunar valves. We used a well-established hemodynamic intervention, outflow tract banding, to alter blood flow in the chicken embryo at Hamburger and Hamilton (HH) stage 18 (~3 days of incubation; Hamburger and Hamilton, 1992), in order to characterize changes in normal myofibril and mitochondrial development induced by increased hemodynamic load *in vivo*. Outflow tract banding increases blood pressure throughout the circulatory system (Tobita et al., 2002; Shi et al., 2013) and blood flow velocities in the constricted region of the outflow tract (Rugonyi et al., 2008; Midgett et al., 2014). Blood pressure and blood flow velocity increases strongly depended on the degree of band tightness (Shi et al., 2013; Midgett et al., 2014) and result in a wide spectrum of heart defects in the chicken embryo (Clark and Rosenquist, 1978; Clark et al., 1989; Hogers et al., 1997; Sedmera et al., 1999; Tobita et al., 2002; Midgett et al., 2017b). We used chicken embryos as a model of human heart development (which is highly conserved among vertebrate species) to allow for ease of accessibility in the egg for surgical manipulation and *in vivo* imaging (McQuinn et al., 2007; Rugonyi et al., 2008; Shi et al., 2013). This work elucidates how increased hemodynamic load detrimentally interferes with normal myofibril and mitochondrial arrangement, which may be fundamental in understanding the origins of CHD. Specifically, this study indicates that normal myocardial and mitochondrial reorganization is one of the initial processes affected by increased hemodynamic load. Banded samples had thicker and more randomly oriented myofibril bundles and mitochondria organized in larger clusters compared to controls. These changes were accompanied by abnormal proteomic expression, possibly implicating the renin-angiotensin system (RAS) in the early response to altered hemodynamic load by banding.

## Methods

### Hemodynamic intervention

Fertilized White Leghorn chicken eggs were incubated blunt end up at 38°C and 80% humidity until stage HH18 (~3 days; Hamburger and Hamilton, 1992), when the heart is a looped tubular structure. Embryo hemodynamics were altered with a 10-0 nylon suture passed under the outflow tract and tied in a knot around the mid-section of the outflow tract to constrict the cross-sectional area. A control group of embryos served as a surgical sham where the suture was passed under the outflow tract but not tightened. Following interventions, eggs were sealed with saran wrap and incubated until further evaluation.

Chick embryos are not considered live vertebrate animals under IACUC and Oregon Health & Science University regulations until they hatch, however we made every effort to minimize the number of embryos needed.

### Band tightness measurement with optical coherence tomography (OCT)

A custom-made optical coherence tomography (OCT) system was used to measure chick embryo band tightness *in vivo* as previously described (Rugonyi et al., 2008; Ma et al., 2010; Liu et al., 2012; Shi et al., 2013). Briefly, the system has a spectral domain configuration with a superluminescent diode centered at 1,325 nm from Thorlabs Inc. (Newton, NJ, USA) and a 1,024 pixel, 92 kHz maximal line-scan rate infrared InGaAs line-scan camera from Goodrich Inc. (Charlotte, NC, USA). It acquired 512 × 512 pixel, 2D B-mode tomographic images at 140 frames per second with <10 μm resolution. Embryo temperature during OCT acquisition was maintained at a normal physiological temperature (38°C) with a thermocouple-controlled heating system. Each banded embryo was imaged immediately before and 2 h after manipulation with OCT to acquire 200 tomographic frames (~3–4 cardiac cycles) of a longitudinal section of the outflow tract in order to measure the change in outflow tract diameter and calculate the degree of band tightness

(1) $$\begin{array}{l} \text{Band tightness}=1-{\text{D}}_{a}/{\text{D}}_{b}, \end{array}$$

where *D*_*a*_ is the maximum external diameter of the outflow tract at the band site after banding, and *D*_*b*_ is the maximum external diameter of the outflow tract at the approximate band site location before banding. The measured band tightness of each banded embryo was used to define the hemodynamic environment, based on our previous characterization of the relationship between the degree of outflow tract band tightness and the specific blood pressure and velocity conditions induced (Shi et al., 2013; Midgett et al., 2014). Band tightness in this study was limited to 30–45% constriction, which corresponds to a relatively constant and large (about 3-fold) increase in wall shear rate/stress (Midgett et al., 2014) and moderate (about 1.5-fold) increase in blood pressure (Shi et al., 2013). After OCT imaging, the eggs were re-sealed with saran wrap and placed back in the incubator.

### FIB-SEM acquisition

Embryos with band tightness between 30 and 45% constriction were collected at HH24 (~24 h after surgical manipulation at HH18), still corresponding to looping tubular stages of heart development. Samples were processed for focused ion beam scanning electron microscopy (FIB-SEM). FIB-SEM imaging was performed on an FEI Helios 660 NanoLab™ DualBeam™. Whole embryos were removed from the egg and immediately immersed in fixative (2.5% paraformaldehyde + 2.5% glutaraldehyde) to maximally preserve and contract the tissue (Damon et al., 2009; Rennie et al., 2014). A portion of the tubular outflow tract containing the endocardial cushions upstream of the banding location was then dissected from the embryo. The fixed tissue was stained and processed as previously described (Rennie et al., 2014), and oriented longitudinally in the resin block face to contain a cross-section of the outflow tract and easily locate the region of interest for imaging. The samples were then processed for FIB-SEM imaging of the myocardium to acquire serial (3D), high-resolution images, as previously described (Rennie et al., 2014). The FIB-SEM system alternated between imaging the block face with a scanning electron beam and milling away 4 nm thick sections of the block face, in order to collect a high-resolution 3D image volume. A maximum of 1000 slices through the myocardium were acquired on a region of interest with a horizontal field width of 20 μm.

### Image analysis and 3D organelle quantification

Amira software (FEI) was used for all FIB-SEM image registration, processing and quantification. First, the acquired image stacks were automatically aligned to reconstruct the 3D image volume. Myofibril bundles, mitochondria, nuclear, and extracellular material were then manually segmented (for consistency) from 500 image stacks and reconstructed, in order to quantify organelle 3D volume and organization.

**Figure 1:**
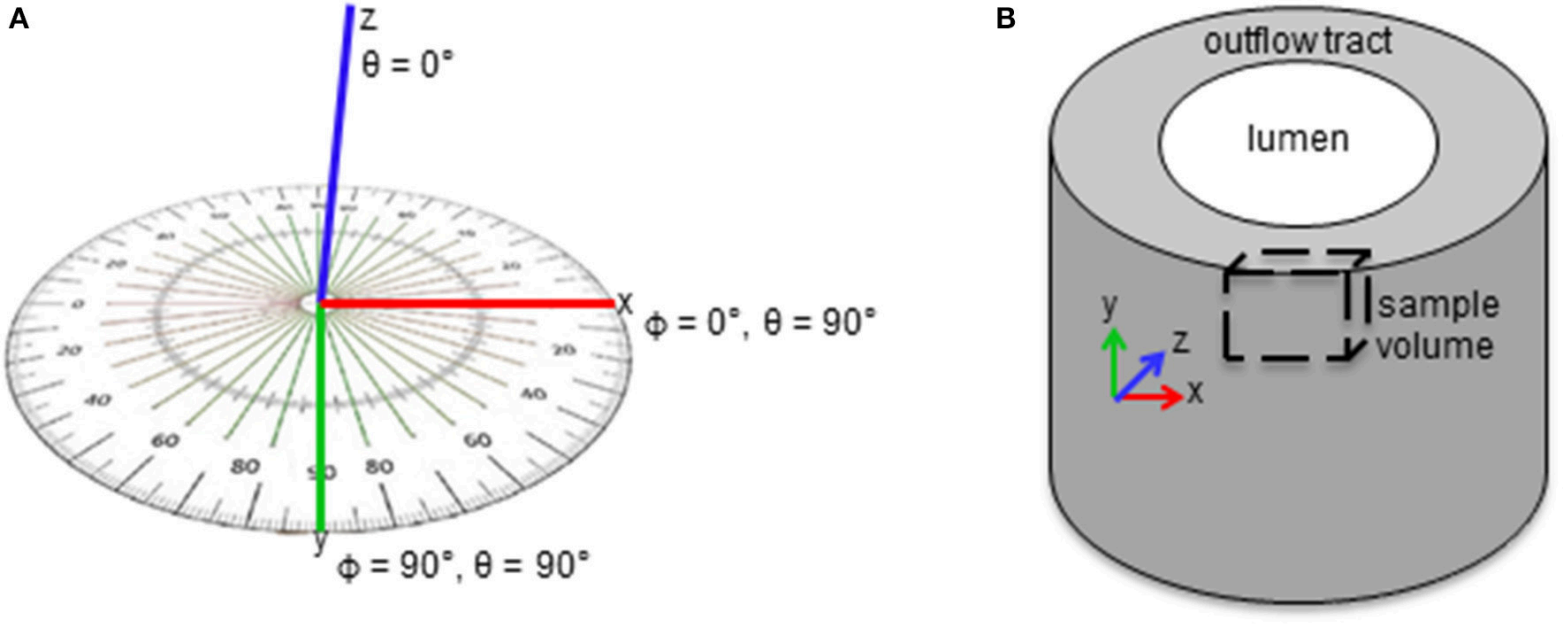
Schematics of myofibril bundle orientation quantification. (A) Angles ϕ and θ were measured for each bundle. **(A)** ϕ and θ are the angles of the fibril bundle in the xy and xz planes in the sample volume, respectively. **(B)** ϕ and θ describe the bundle orientation in the circumferential and radial planes of the outflow tract, respectively.

Myofibril and mitochondrial volume fractions in the cell cytoplasm (instead of total cell volume) were used to quantify organelle volume, which accounted for differences in sample nuclear volume that depended on the specific region of interest. Cytoplasm volume was calculated by subtracting the volume of extracellular and nuclear material from the total sample volume. Myofibrils were additionally quantified by bundle thickness and orientation within the outflow tract. Myofibril bundle thickness was used as a measure of the extent of myofibril development, and quantified as the average of measured lengths of Z-lines of all bundles in each sample. Myofibril bundle orientation was quantified in terms of angles ϕ and θ (see Figure 1), where ϕ is the angle of the fibril bundle in the xy plane (circumferential plane of the outflow tract, with ϕ = 90° indicating longitudinal alignment and ϕ = 0° indicating circumferential alignment), and θ is the angle of the fibril bundle in the xz plane (radial plane of the outflow tract, with θ = 0° indicating radial alignment and θ = 90° indicating circumferential or longitudinal alignment). Both orientation angles were measured for all myofibril bundles defined between Z-lines for each sample. Angle values were scaled so each measure ranged from 0 to 90°, to capture the fact that myofibrils with 5° and 175° angle orientation values in a single plane are similarly oriented with respect to longitudinal, circumferential or radial directions. Since the exact orientation of the outflow tract tissue within the resin block for FIB-SEM imaging cannot be controlled, absolute myofibril orientation angle values could not be compared. Instead, the distribution of the orientation angles within each sample was used to analyze the overall organization pattern.

### Mass spectrometry

In separate experiments, after surgical manipulations at HH18, outflow tracts were dissected from the embryo at HH24 and rinsed in ultrapure water. There were a total of 5 banded embryo samples (30–45% band tightness) and 5 sham control samples included in these experiments, with each sample consisting of 8 pooled outflow tracts (40 hearts per treatment distributed into 5 samples). Pooling outflow tracts was necessary to reach the minimum mass required for the mass-spectrometry analysis. Samples were then processed as described before (Midgett et al., 2017a). Briefly, samples were first lysed and sonicated, and lysate protein content determined using a BCA assay (Thermo Scientific) and BSA standard. After further sample processing (involving sample reduction, alkylation, trypsinization, incubation and centrifugation), tandem mass tagging (TMT) reagent (Thermo Scientific) was added to each sample and incubated at room temperature for 1 h. A short single liquid chromatography–mass spectrometry (LC-MS) analysis was then performed to check for complete labeling and to determine the relative summed reporter ion intensities of each sample. This normalization allowed confirmation of >95% TMT labeling and generated preliminary summed reporter ion intensities for each TMT channel. The 10 samples were then loaded in each channel after adjusting sample volumes so that each sample would yield approximately equal summed reporter ion intensities in the final mass-spectrometry analysis. Tandem mass spectrometry data was collected using an Orbitrap Fusion Tribrid instrument configured with an EasySpray NanoSource (Thermo Scientific). Survey scans were performed in the Orbitrap mass analyzer, and data-dependent MS2 scans in the linear ion trap using collision-induced dissociation following isolation with the instrument's quadrupole. Reporter ion detection was performed in the Orbitrap mass analyzer using MS3 scans following synchronous precursor isolation of the top 10 most intense ions in the linear ion trap, and higher-energy collisional dissociation in the ion-routing multipole.

### TMT data analysis

Raw instrument files were processed using Proteome Discoverer version 1.4.1.14 (Thermo Scientific) with SEQUEST HT software and a Gallus gallus Ensembl database (release 85) containing 16,187 sequences. Searches were configured with static modifications for the TMT reagents (+229.163 peptide N-terminus and K residues) and iodoacetamide (+57.021 C residues), variable oxidation (+15.995 M residues), parent ion tolerance of +/− 1.25 Da, fragment ion tolerance of 1.0 Da, monoisotopic masses, and trypsin cleavage (max 2 missed cleavages). Searches used a reversed sequence decoy strategy to control peptide false discovery and identifications were validated using Percolator software. Only peptides having mass errors <20 ppm, and matching only one protein entry in the database were used. Search results and TMT reporter ion intensities were exported as text files and processed using in-house scripts. After excluding the highest and lowest reporter ion intensities, an average intensity >500 was required for the remaining reporter ions to exclude the analysis of peptides with insufficient signal intensity. Reporter ion intensities for peptides assigned to each protein were then summed to create an abundance measurement for each protein across the 10 samples simultaneously analyzed.

### Statistical analysis

FIB-SEM samples and TMT samples were selected with a relatively small range of band tightness (30–45%), and therefore differences between samples with varying band tightness were not analyzed. Rather, analysis was performed to determine differences between control and banded samples. Statistical significance between banded and control groups using FIB-SEM image analysis was determined with a two-sample Student's *t*-test, assuming significance with two-tail *p* < 0.05 unless otherwise noted.

Differential protein abundance was determined in the TMT experiment by comparing the summed peptide reporter ion intensities for each protein across samples (5 control and 5 banded) using the R software (v 3.1.1) package edgeR (Robinson et al., 2010), which performed data normalization, fold changes, determination of individual protein *p*-values, Benjamini–Hochberg multiple test correction to calculate false discovery rates (FDR or *q*-value) during tests for differential abundance for each protein compared between banded and control samples. These data was used in comparing changes in abundance of specific proteins.

### Pathway analysis

A pathway analysis of proteomics data was performed using Ingenuity Pathway Analysis (IPA) software (Qiagen Bioinformatics). Protein intensities quantified for each sample (5 control and 5 banded) were used to compute differential protein abundances and protein fold ratios (and their logarithms) between banded and control treatments, as well as *p*-values, and false discovery rates (*q*-values), as described above using edgeR. We then filtered the data for low intensity values and peptide counts per protein: we discarded protein measurements with low intensity (with at least two of the samples having a low intensity reading <4,000) and proteins obtained using only one peptide. That is, for analysis we kept only proteins that were relatively abundant and measured with accuracy. This rendered 3838 proteins of which 2,544 could be mapped to known proteins/genes in IPA. The filtered data (protein log fold ratios, and *p*-values) were used as input for the IPA analysis (provided as Supplementary file “Data Sheet 1,” together with the IPA dataset “Data Sheet 2”). We performed the analysis using the Ingenuity Knowledge Base, a pathway database in IPA, including direct and indirect relationships, considering genes only, endogenous chemicals in network analysis, and with experimentally observed confidence. The analysis was further performed using all node types, available data sources, and all species pathways available, but no mutations considered. Analysis included data from all tissues and cell lines, and was restricted to proteins featuring significant differences in banded and control samples (*p* < 0.1, total 948 proteins analyzed). IPA rendered a list of affected canonical pathways with their *p*-values, which measure statistically significant overlap between the dysregulated input-gene dataset and genes in the pathway, and was calculated using Fisher's exact test. Regulation direction, however, is not considered into the calculation of the pathway *p*-value, and is included instead in the z-scores. The z-score in IPA is defined as

(2) $$\begin{array}{l} \text{z}-\text{score}=\left({\text{N}}_{+}-{\text{N}}_{-}\right)/{\text{N}}^{1/2}, \end{array}$$

where N is the number of detected dysregulated proteins in the pathway, N_+_ and N_−_ are proteins that have changed abundance in the direction of pathway activation and inhibition (according to literature-derived regulation direction incorporated in IPA), respectively. Pathway z-scores therefore quantify activation or inhibition of pathways based on the differential abundance of detected proteins in the pathway.

## Results

### Myofibril and mitochondrial maturation is active at HH24

**Figure 2:**
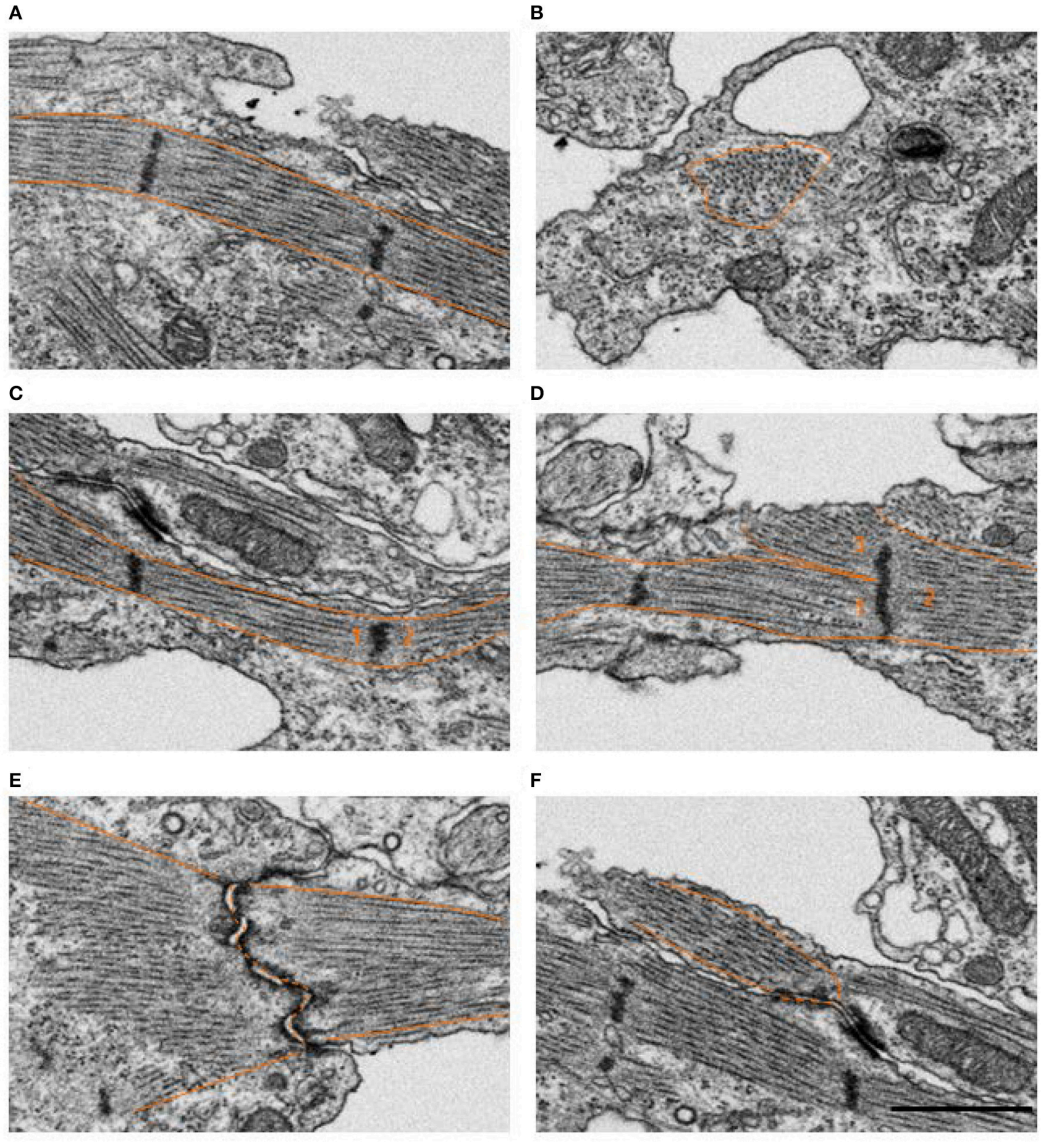
Example FIB-SEM image showing myofibrils in control outflow tract myocardium tissue. Several myofibril orientations are displayed, where the tissue mostly consisted of longitudinally oriented bundles arranged circumferentially around the outflow tract **(A)** compared to less frequent radially oriented bundles **(B)**. The majority of z-bands only had a single myofibril bundle extending from either side **(C)**, however, samples also contained a few examples of an immature configuration where 3 or more fibril bundles radiated from the same z-band center **(D)**, as numbered above. Most myofibrils inserted into intercalated disks at nearly right angles as they do in mature tissue **(E)**, while some oblique insertions were still visible in control tissue **(F)**. Scale bar = 1 μm.

**Figure 3:**
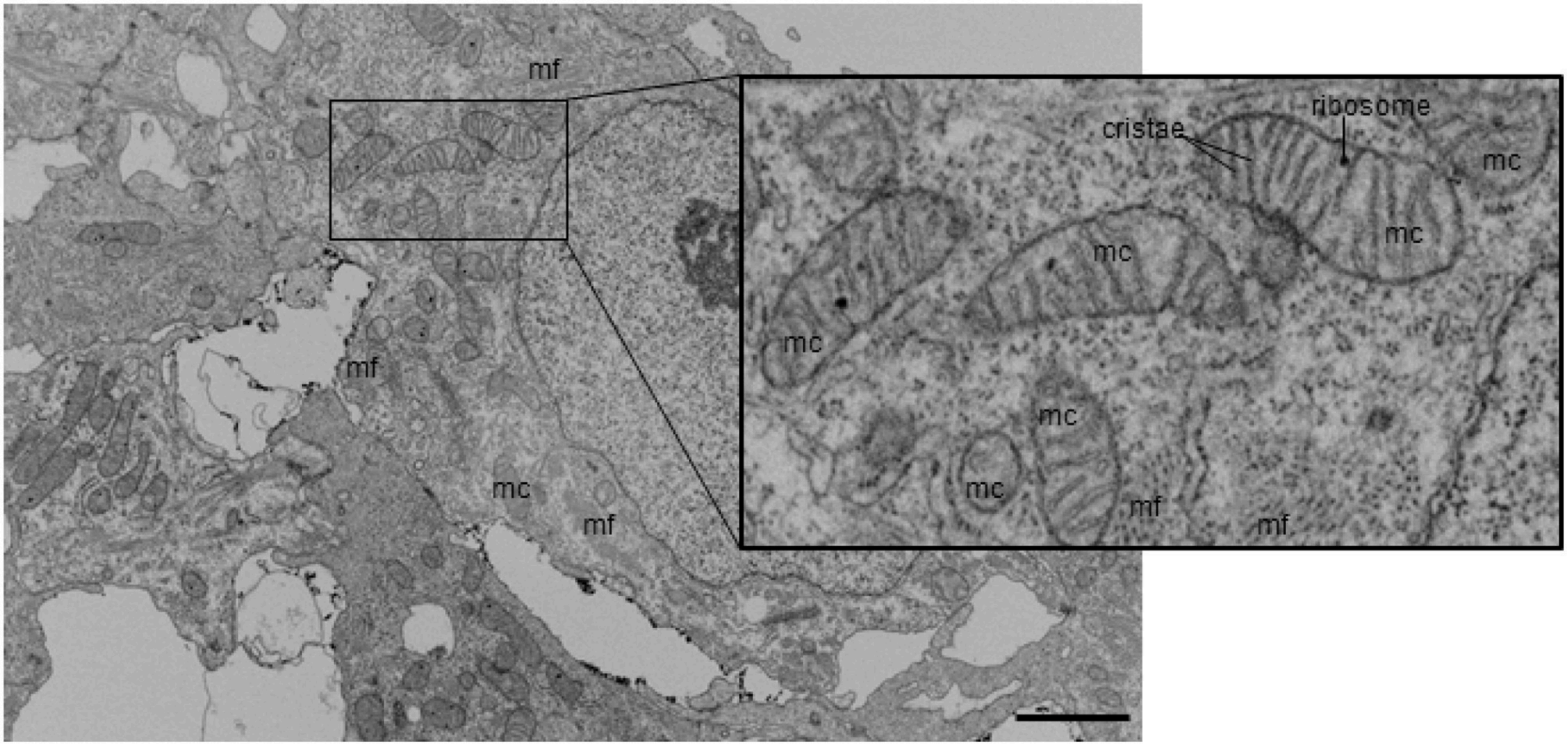
Example FIB-SEM image showing mitochondria among myofibrils in control outflow tract myocardium tissue. Myofibrils are arranged in both circumferential and radial orientations. Mc, mitochondria; mf, myofibril. Scale bar = 5 μm.

FIB-SEM processing acquired 3D image data from the myocardium layer of the heart at HH24 with excellent myofibril and mitochondrial ultrastructural detail. Control outflow tract myocytes contained immature myofibril structures (Figure 2), indicating that the development of the outflow tract heart muscle is still ongoing at this stage, as expected. Immature myofibril organization was characterized by the overall lack of myofibril alignment (see Figures 2A,B) and instances where more than one fibril radiated from the same z-band center (see Figure 2D). In addition, while most myofibrils inserted into intercalated disks at nearly right angles as they do in mature tissue, some oblique insertions were still visible in control tissue (see Figure 2F). Mitochondria were scattered throughout the cytoplasm with clear walls, cristae, and ribosome structures at this stage of development (Figure 3). Control tissue contained an array of mitochondrial shapes ranging from very long and tubular-like to almost spherical, indicating that mitochondrial maturation is in an active fusion/fission maturation phase. Mitochondria appeared in small groups that were distributed throughout the cytoplasm and around myofibril bundles.

### Myofibril and mitochondrial volume fraction is unchanged after increased hemodynamic load

**Figure 4:**
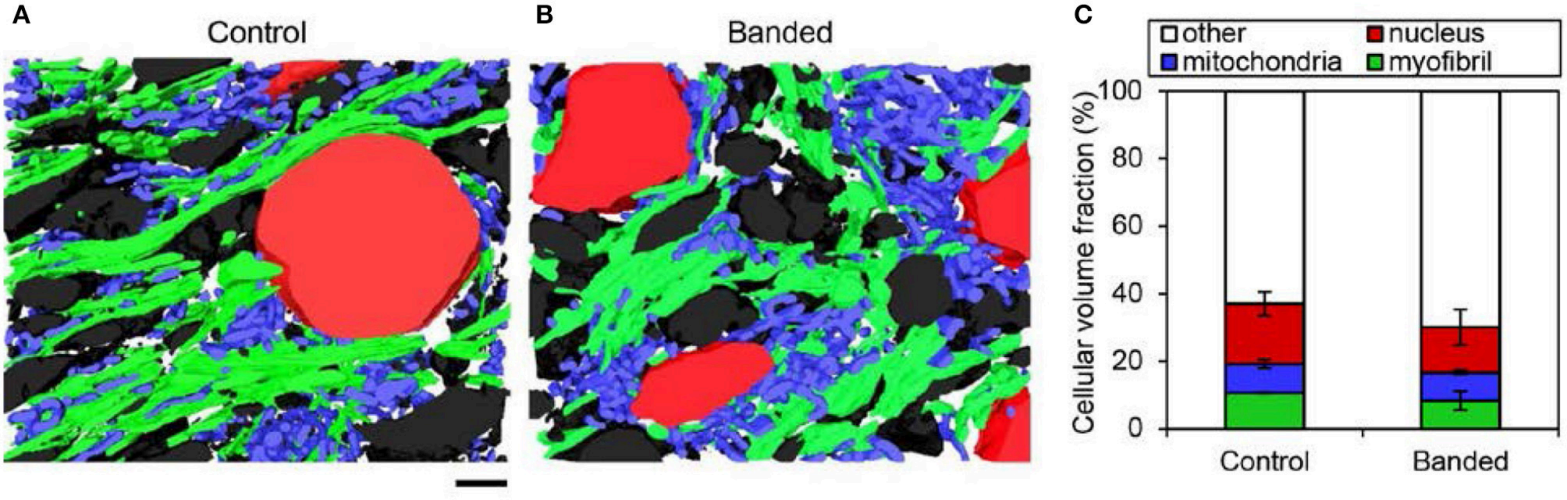
FIB-SEM organelle volume quantitation summary. Example 3D myocardium segmentation reconstructions from a control **(A)** and banded **(B)** embryonic heart with myofibril, mitochondrial, nuclear, and extracellular material displayed in green, blue, red, and black, respectively. **(C)** Average cellular volume fraction quantification from 3D reconstructions (*n* = 3 per group), where there were no significant differences of any cellular component between banded and control samples. Scale bar = 2 μm.

To quantify alterations in myofibril and mitochondrial volume after outflow tract banding, these organelles were segmented from high-resolution FIB-SEM image volumes of myocardial outflow tract tissue. Myofibril and mitochondrial volume fraction in the cell cytoplasm were calculated from the 3D reconstructions of the segmented images (Figure 4). The myofibril-cytoplasm volume fraction did not significantly vary between control and banded samples (12.9 ± 0.3 vs. 9.6 ± 2.8%, in control and banded, respectively; *p* = 0.17, *n* = 3). Similarly, mitochondrial-cytoplasm volume fraction was comparable in both groups (10.5 ± 1.8 and 9.7 ± 1.4% in control and banded; *p* = 0.56, *n* = 3).

### Increased hemodynamic load triggers myofibril reorganization and mitochondrial misalignment

**Figure 5:**
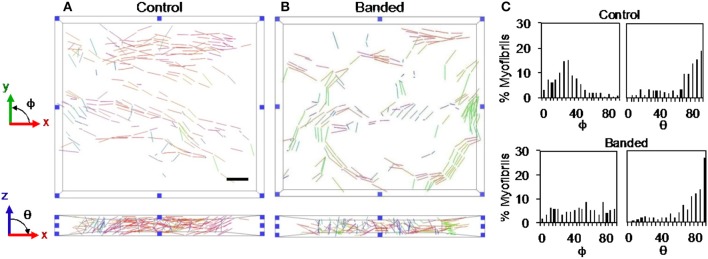
Example myofibril bundle orientation display where each bundle is shown colored based on the orientation in the xy and xz planes for a control **(A)** and a banded **(B)** embryo. **(C)** Example myofibril bundle orientation angle histograms for a control and banded embryo. Scale bar = 2 μm.

3D FIB-SEM reconstructions of myofibril bundle segmentations were used to calculate fibril bundle thickness and orientation. While myofibril volume fraction and orientation in the radial plane (x-z plane in Figure 1) did not significantly change after banding, fibril bundles were more clustered; orientation in the circumferential plane (x-y plane in Figure 1) was more distributed, and bundles were thicker following increased hemodynamic load. Three-dimensional reconstructions from the FIB-SEM samples (Figure 4) qualitatively show that the segmented myofibril material was more clustered in banded samples compared to controls where the myofibril material was distributed throughout the cells. Myofibril bundle orientation was quantified in terms of angles ϕ and θ (see Figure 1), where ϕ is the angle of the fibril bundle in the x-y plane (circumferential plane of the outflow tract sample), and θ is the angle of the fibril bundle in the x-z plane (radial plane of the outflow tract sample). The ϕ myofibril angles in control myocardium concentrated in the region of low ϕ with an average of 42.6% of myofibrils within a sample concentrated within a 20 degree range of ϕ, indicating more predominant fibril alignment in the circumferential direction (ϕ = 0). In contrast, the fibril orientation in this plane in banded samples was more distributed and contained fibers that pointed in both circumferential and longitudinal directions of the outflow tract myocardium (Figure 5), with an average of only 24.7% of myofibrils concentrated within a 20 degree range of ϕ (ϕ 0–20°). The distribution of θ angles in the myofibrils was similar between control and banded samples and indicated a preferential distribution of the fibers in the circumferential direction of the outflow tract (θ = 90°).

**Figure 6:**
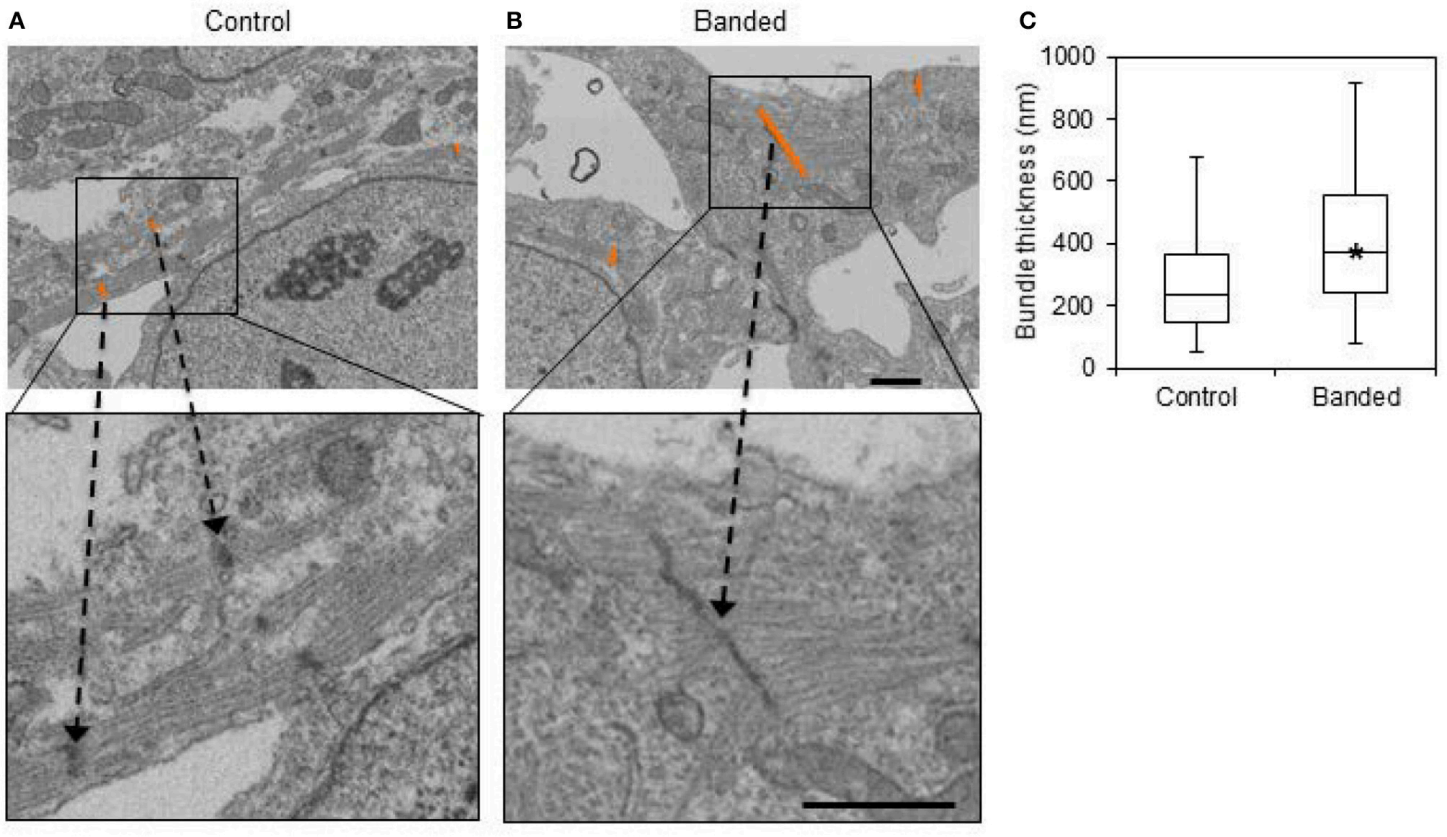
Example FIB-SEM images from a control **(A)** and a banded **(B)** embryo, with example bundle thickness measurement lengths at z-bands shown in orange. **(C)** Bundle thickness quantification box plots displaying the average, with boxes that mark the upper and lower quartiles and whiskers that mark the maximum and minimum values. The asterisk designates a significantly higher bundle thickness average in banded samples compared to controls (*p* < 0.05, *n* = 3). Scale bar = 1 μm.

Additionally, the average bundle thickness across samples in each group was statistically larger in banded samples (*p* << 0.01, *n* = 3). Box plots show that there was a larger range of bundle thicknesses in banded samples, indicating that these samples had both small and abnormally large bundles (Figure 6).

Despite unchanged mitochondrial volume fraction in banded samples compared to controls, qualitative differences in the mitochondrial arrangement were displayed in FIB-SEM 3D reconstructions (Figure 4). Control and banded tissue contain an array of mitochondrial shapes ranging from very long and tubular-like to almost spherical, indicating that mitochondrial maturation is active. Mitochondria, however, appeared more clustered in banded samples where each sample showed ~2–3 main groups of mitochondria that were largely void of myofibrils (see Figure 4). While there were still clusters of mitochondria in control samples, they clustered in smaller groups and were overall more distributed throughout the myofibrils.

### Increased hemodynamic load triggers myofibril and mitochondrial proteomic response

**Figure 7:**
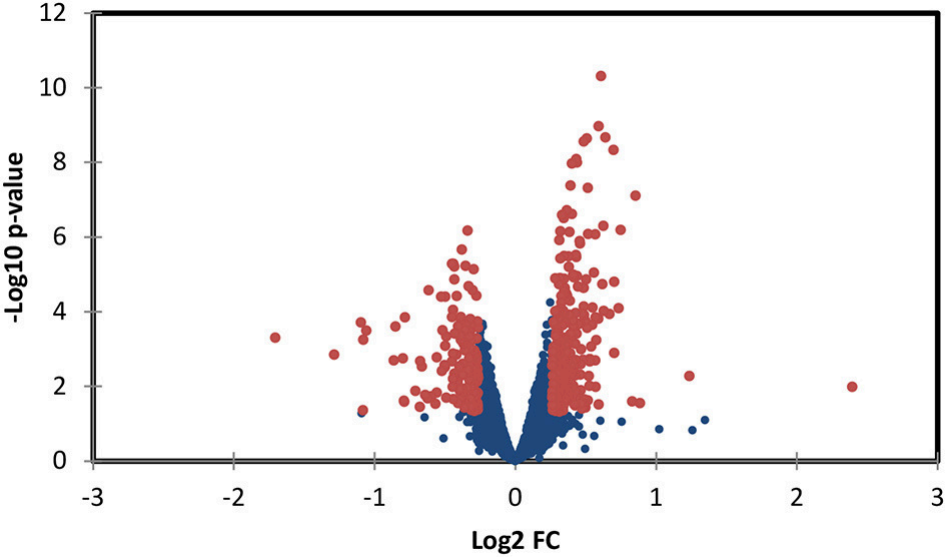
Volcano plot of proteomics data, depicting protein data *p*-values vs. fold change (FC). Red points represent data points with *p* < 0.05 and a fold rate >1.2; proteins associated with these points are listed in the supplementary file “Data Sheet 3.”

**Figure 8:**
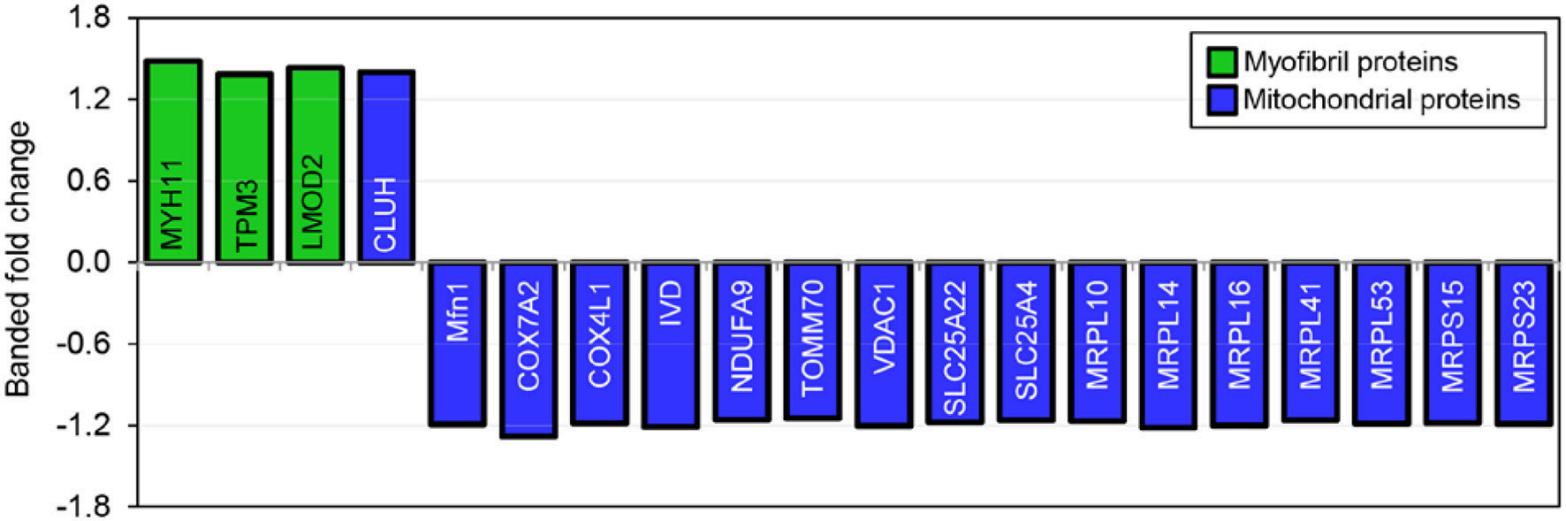
Fold changes in mass-spectrometry measured protein abundances in banded samples compared to controls. Proteins associated with myofibril and mitochondrial proteins are shown, with positive and negative fold changes representing protein upregulation and downregulation, respectively. Protein abbreviations are listed in Tables 1, 2.

TMT based mass spectrometry provided a proteome-wide assessment of changes in the relative abundance of specific proteins induced by increased hemodynamic load. Specifically, we first focused on examining changes in proteins known to be markers of myofibril and mitochondrial reorganization. A total of 5,330 proteins were detected in the outflow tract samples, where the abundance of 606 proteins significantly differed between banded and control samples (false discovery rate, *q* < 0.1, *n* = 5). The full proteomics data set was deposited in the PRIDE repository database (ProteomeXchange Consortium), with dataset identifier PXD005362. A volcano plot of the data is shown in Figure 7, with point in red listed in the Supplementary file “Data Sheet 3.” Of the proteins that were significantly up or downregulated, 3 proteins were related to myocardial contraction, and 17 were directly related to mitochondrial form and function. All proteins had at least 2 peptide-spectrum matches with reporter ion signal used in quantitation among our samples. The main protein changes included components of myocardial thin filament assembly and function; and mitochondrial maintenance, organization, and the electron transport system (Figure 8). Table 1 lists changes in proteins related to myocardial contractile proteins and Table 2 lists changes in cardiac mitochondrial proteins. Each table gives the protein fold changes between control and banded samples and lists each protein function in terms of myocardial reorganization. Most fold-changes in protein abundances are relatively small (1.1- to 1.5-fold), which may reflect a modulation of responses by banding and the relatively short intervention time span (~24 h). Overall proteomics data indicates an increase in proteins associated with myocardial contraction and mitochondrial clustering; and a decrease in proteins associated with mitochondrial fusion, electron transport, and metabolic function.

**Table 1:** Myocardial contractile proteins.

	**Banded fold change**	***q*-value**	**Function**
Myosin, heavy chain 11, smooth muscle (MYH11)	+1.5	0.04	Contractile protein of smooth muscle cells (England and Loughna, 2013).
Tropomyosin 3 (TPM3)	+1.4	0.03	Member of the tropomyosin family of actin-binding proteins that are central to the control of calcium-regulated thin filament function and striated muscle contraction (Rajan et al., 2010; Bai et al., 2013).
Leiomodin 2 (LMOD2)	+1.4	<0.01	Actin-binding protein that promotes the regulation of striated muscle thin filament assembly (Tsukada et al., 2010; Pappas et al., 2015).

*q-value: false discovery rate adjusted p-value*.

**Table 2:** Cardiac mitochondrial proteins.

**Protein**	**Banded fold change**	***q*-value**	**Function**
Clustered Mitochondria (CluA/CLU1) Homolog (CLUH)	+1.4	<0.01	mRNA-binding protein involved in proper cytoplasmic distribution and clustering of mitochondria, that plays a role in mitochondrial biogenesis (Zhu et al., 1997; Fields et al., 1998; Gao et al., 2014).
Mitofusin 1 (MFN1)	−1.2	0.08	Essential protein required for mitochondrial fusion which affects mitochondrial morphology (Chen et al., 2003).
Cytochrome c oxidase subunit VIIa polypeptide 2 (COX7A2)	−1.3	0.03	Terminal oxidative phosphorylation Protein complex that plays a role in the mitochondrial electron transport chain (Hüttemann et al., 2012).
Cytochrome c oxidase subunit IV isoform 1 (COX4I1)	−1.2	0.02	
isovaleryl-CoA dehydrogenase (IVD)	−1.2	0.03	Matrix enzyme that is important in mitochondrial fatty acid oxidation (Hale et al., 1985).
NADH dehydrogenase (ubiquinone) 1 alpha subcomplex 9 (NDUFA9)	−1.2	0.03	Protein in the inner mitochondrial membrane that is the first protein complex in the electron transport chain (Hu et al., 2009).
Translocase of outer mitochondrial membrane 70 (TOMM70)	−1.1	0.03	Import receptor of the outer mitochondrial Membrane that helps mediate the transition of precursor proteins from ribosomes to mitochondria (Sokol et al., 2014).
Voltage-dependent anion channel 1 (VDAC1)	−1.2	0.01	Channel that provides exchange of metabolites and ions Across the outer mitochondrial membranes that are necessary for electron transport (Das et al., 2012).
Solute carrier family 25, member 22 (SLC25A22)	−1.2	0.04	Mitochondrial carrier protein that transports glutamate through the inner mitochondrial membrane (Walker and Runswick, 1993).
Solute carrier family 25, member 4 (SLC25A4)	−1.3	0.03	Mitochondrial carrier protein that transports adenine nucleotides through the inner mitochondrial membrane (Walker and Runswick, 1993).
Mitochondnrial ribosomal protein L10 (MRPL10)	−1.2	0.03	Mitochondrial ribosomal proteins are involved in mitochondrial translation, biogenesis, and maintenance and are critical for healthy mitochondrial function (Sylvester et al., 2004).
Mitochondnrial ribosomal protein L14 (MRPL14)	−1.2	0.03	
Mitochondnrial ribosomal protein L16 (MRPL16)	−1.2	0.01	
Mitochondnrial ribosomal protein L41 (MRPL41)	−1.2	0.04	
Mitochondnrial ribosomal protein L53 (MRPL53)	−1.2	0.02	
Mitochondnrial ribosomal protein S15 (MRPS15)	−1.2	0.04	
Mitochondnrial ribosomal protein S23 (MRPS23)	−1.2	0.01	

*q-value, false discovery rate adjusted p-value*.

### Increased hemodynamic load affects pathways related to mitochondrial function and stress response

**Figure 9:**
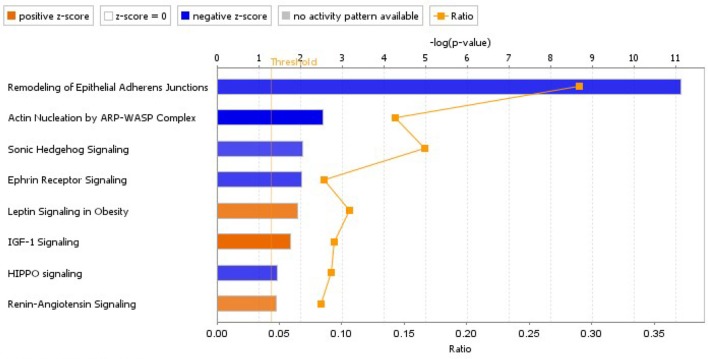
Significantly inhibited and activated pathways due to banding treatment, based on proteomics data. The analysis was performed in IPA, using data from proteins that changed abundance significantly (*p* < 0.1; 948 proteins included in the analysis), and considering information from all tissues/cell lines. Pathways plotted have *p* < 0.05 and z-score absolute value >2 (blue denotes inhibition and orange activation). The plotted ratio shows the ratio of the number of dysregulated molecules in our analysis data set that corresponded to the pathway, vs. the total number of molecules in the canonical pathway.

A pathway analysis of proteomics data using IPA software resulted in a list of canonical pathways differentially affected by banding. We first selected pathways that were significantly affected (pathway *p* < 0.05), and that were either significantly activated or inhibited (z-score absolute value > 2.0), when performing the analysis using only dysregulated proteins with *p* < 0.1 (see Figure 9). A detailed graphical depiction of pathways of interest is shown in the Supplementary file “Presentation 1;” while lists of dysregulated genes per pathway are shown in the Supplementary file “Data Sheet 4.” Note, however, that in general the ratio of detected dysregulated molecules per pathway with respect to pathway molecules is small, likely due to limitations in the proteomics approach that did not detect less abundant proteins as well as our filtering prior to analysis. The inhibition of the “Remodeling of Epithelial Adherens Junctions” pathway is likely related to the changes in endothelial mesenchymal transition (EMT) that we have reported before (Midgett et al., 2017a), where there is an increased cell density in outflow tract cardiac cushions with banding vs. control, presumably due to increased EMT.

Except for the RAS pathway, other pathways selected exhibited changes in abundance in downstream proteins, but not in the proteins and receptors initiating the signaling cascades. While these pathways regulate cell behaviors (e.g., cell migration and proliferation) and could indeed be indispensable for cardiac development (e.g., Sonic Hedgehog Signaling), validation would be required to show pathway activation/inhibition in response to banding.

In the RAS pathway, we found increase in abundance of upstream regulators. In particular, we found an increase in angiotensinogen (AGT; fold change 1.3, *p* < 2 · 10^−4^) and the angiotensin I converting enzyme (ACE; fold change 1.2, *p* < 0.02). These upstream effectors initiate signaling through angiotensin II (Ang II) after converting AGT into Ang I and then Ang II. Downstream proteins, in addition, were also affected. In particular STAT3 increased 1.2-fold in banded hearts compared to controls (*p* < 0.002). Nevertheless, due to the relatively low number of proteins detected in the RAS pathway, validation of pathway activation needs to be performed in future research. RAS is known to play an important role in the response of the fetal and mature heart to pressure, and its involvement could be pivotal in heart development.

Other canonical pathways highlighted in our analysis were the “Mitochondrial Dysfunction,” “EIF2 Signaling,” and “Oxidative Phosphorylation” pathways (all with *p* << 0.001). Both the *Mitochondrial dysfunction* and *Oxidative phosphorylation* pathways are related to regulation of mitochondrial function and showed a reduction in components of the mitochondrial complexes I, IV, and V, as well as a (a weaker) reduction in Complex III. While z-scores for these pathways were not available since there was not enough information on the IPA database to determine pathway activation status, the Molecule Activity Predictor (MAP) tool available in IPA predicted decrease of ATP production associated with these pathways. The *EIF2 signaling* pathway, in contrast, was activated (z-score 1.9) and it is associated with global protein synthesis and regulation of mRNA. MAP applied to the *EIF2 signaling* pathway predicted increased cardiac protection, as well as increase amino acid transport and synthesis. Interestingly, and in connection with mitochondrial function, we found significant decreases in the cAMP-responsive element binding 1 (CREB1) protein (fold change 0.8, *p* < 0.003); and in the mitochondrial transcription factor A (TFAM; fold change 0.9, *p* < 0.02).

## Discussion

Myofibril and mitochondrial organization in the myocardium of the early embryonic outflow tract was analyzed after increasing hemodynamic load via a surgical intervention. Outflow tract banding increases blood pressure throughout the circulatory system (Tobita et al., 2002; Shi et al., 2013) and blood flow velocities in the outflow tract (Rugonyi et al., 2008; Midgett et al., 2014). Increase in blood pressure and flow velocity (and the associated increase in wall shear rates/stresses) are dependent on the degree of band tightness (Shi et al., 2013; Midgett et al., 2014). Our previous investigations showed that peak (maximum) blood flow velocity increased with band constriction up to ~35% band tightness, above which it plateaued and decreased, while peak ventricular pressure increased approximately exponentially with constriction. The band constriction range of 30–45% used for this study corresponds to maximal increase in flow velocity induced by banding (~3-fold increase in wall shear rates), while blood pressure increase was more moderate (~1.5-fold from control). Furthermore, these blood flow conditions described by the degree of band constriction lead to specific abnormal cardiac structure and function in later stages of development, including ventricular septal defects and double outlet right ventricle defects (Midgett et al., 2017b). This study describes the earliest remodeling effects involved in the initiation of harmful tissue remodeling that eventually lead to cardiac defects. While several authors studied the response of the myocardium ventricle to load, in this work we chose the heart outflow tract since ultimately most cardiac defects in humans have their origins in this cardiac segment.

Myofibril and mitochondria were segmented from FIB-SEM image volumes, in order to reconstruct 3D organelle structures and analyze the architecture of mitochondrial-myofibril networks. Changes in organelle volume fraction and organization were evaluated in outflow tract banded and control myocardial tissue samples to elucidate the effects of increased hemodynamic load on myofibril and mitochondria. While altered blood flow likely triggers spatially and temporally dependent effects along the length of the tubular heart over the course of development, the FIB-SEM samples in this study were from a single location upstream of the banding site near the outflow tract cushions at a single embryonic stage ~24 h after the hemodynamic intervention. Therefore, our study highlights early changes in the outflow tract tissues in response to banding that likely affects further heart development and malformation at later stages.

Even though myofibril and mitochondrial volume fraction is unchanged after increased hemodynamic load, altered myofibril and mitochondrial organization suggest that abnormal hemodynamics induced by banding interferes with the normal myocardial cell maturation process. Unchanged organelle volume fractions after banding is consistent with a study by Clark et al. that found similar organelle proportion of myofibrils and mitochondria, DNA-to-protein ratio, and myocyte area after outflow tract banding at HH21 and concluded that measured increases in ventricular weights were due to myocyte hyperplasia (Clark et al., 1989). Nevertheless, banded samples had both thicker myofibril bundles that were more randomly aligned in the myocardium, and mitochondria that were organized in larger clusters compared to controls. While the number of FIB-SEM samples in this study was small because of the limitations of the extensive imaging and analysis required (*n* = 3), these results taken together with the proteomic data indicate an organizational myofibril and mitochondrial response to increased hemodynamic load.

Although, previous studies have characterized myofibril morphology during development, the mechanisms of myofibril formation and alignment are still poorly understood (Sanger et al., 2005). It has been postulated, however, that fibrils align in specific regions of the heart in response to mechanical contraction stresses that increase throughout development in chicken embryos (Manasek, 1970). Raeker et al. suggested that fibril bundles slide to align z-disks as well as dismantle and reassemble to aid in bundling small myofibril clusters to form larger bundle units in skeletal muscle in zebrafish. Further, they concluded that degradation in myofibril organization and reorganization of bundles during development seemed to occur when the offset between z-disks was too large to permit intact integration (Raeker et al., 2014). Differences in ϕ orientation angle distributions in our samples may be due to similar shifting of myofibrils to cluster and align, which results in thicker bundles. These changes in myofibril organization and size suggest that increased hemodynamic load induced by banding may accelerate the normal myocardial maturation process.

FIB-SEM reconstructions from control samples showed mitochondria that appear in small groups that were distributed throughout the cytoplasm and around myofibrils, while mitochondria in banded samples mainly clustered in ~2–3 large groups per sample that were largely void of myofibrils. The large mitochondrial clusters in banded samples were arranged around the nuclei, which is consistent with a progressed stage of mitochondrial organization in embryonic development. Mitochondria are normally scattered throughout the myocyte in early embryonic stages, but as the myofibrils become more dense, mitochondria aggregate around the poles of the nucleus and between myofibrils, and ultimately arrange in a lattice organization throughout the myofibrils (Brook et al., 1983; Vendelin et al., 2005; Chung et al., 2007). Changes in the mitochondrial organization after outflow tract banding indicate that increased hemodynamic load causes mitochondria to conform to a more mature configuration.

Mass-spectrometry was used to detect global changes in protein abundances after banding to reflect the overall dynamic regulation balance between transcription, localization, modification, and programmed destruction of protein during myocardial reorganization in response to altered hemodynamic load. The proteomic analysis corroborates the FIB-SEM evaluations, where we found that increased hemodynamic load triggers a proteomic response corresponding to key myofibril and mitochondrial functional proteins (see Figure 8 and Tables 1, 2). Altered myofibril proteins included components of thin filament assembly and function. Proper contraction of overlapping arrays of actin-containing thin filaments and myosin-containing thick filaments in cardiomyocytes requires appropriate filament alignment. Accordingly, their orientations, spacing, and lengths throughout development are highly regulated by several proteins (Tsukada et al., 2010). For example, Tropomyosin 3 stabilizes filamentous actin and coordinates the actomyosin interaction (Rajan et al., 2010; Bai et al., 2013), while leiomodin 2 is associated with the promotion of actin assembly and thin filament elongation (Pappas et al., 2015). Myosin heavy chain 11, tropomyosin 3, and leiomodin 2 proteins were upregulated in banded samples compared to controls, indicating that these proteins likely work in concert to help produce the changes in myofibril bundle orientation distributions and increased bundle thickness detected with the 3D FIB-SEM analysis.

Analysis of mass-spectrometry data combined with pathway analysis, revealed increases in the abundance of proteins associated with the RAS pathway with banding. In adults, RAS is a key regulator of blood pressure (Te Riet et al., 2015), with its upregulation in the heart leading to cardiac fibrosis and heart failure (Schmieder et al., 2007; Knight et al., 2012). The role of RAS in early cardiac development, however, has been less studied and still needs to be fully elucidated (Price et al., 1997). Our proteomics analysis revealed upregulation of AGT and ACE after 24 h of banding. Activation of the RAS pathway starts with conversion of AGT to angiotensin I (Ang I) by renin, and then Ang I is converted to Ang II by ACE. Angiotensin II then activates the angiotensin receptors 1 or 2 (AT1R or AT2R). In the adult heart, Ang II acts predominantly through AT1R inducing myocardial hypertrophy and fibrosis; with increased hemodynamic load leading to increase Ang II levels (Price et al., 1997). In neonatal pigs, Ang II is required for normal cardiac development (Beinlich and Morgan, 1993; Beinlich et al., 1995). In studies using rats, embryos were allowed to develop to embryonic day 9.5 *in vivo* (tubular heart stages) and then they were cultured for 48 h. Addition of Ang II in the cultured media resulted in increased myofibril development in the heart. Ang II treated hearts exhibited more advanced sarcomere and Z-band organization, which resembled the increased myofibril development that we observed in our banded hearts with respect to controls. Thus our data, in view of previous studies, may imply that myofibril reorganization after increased hemodynamic load in banded hearts may be mediated by RAS. While this relationship will need to be further validated, especially given the low number of proteins in RAS detected by the proteomics analysis, it is certainly an exciting possible direction of research.

We also found increased abundance of STAT3 in banded hearts vs. control hearts. STAT proteins are expressed in the mature heart, and in particular STAT1 and STAT3 play roles in ischemic heart disease, with STAT1 contributing to the decrease of myocardial cells by increasing apoptosis, while STAT3 being cardioprotective by decreasing reactive oxygen species, but related to early remodeling of the heart that then leads to heart failure (Knight et al., 2012). In our embryonic banded heart outflow tracts, STAT3 was increased compared to controls, but STAT1 and STAT5B, while detected in the proteomics analysis, did not change significantly with banding. STATs are recruited and activated by the Janus kinase (JAK) family, and translocate to the nucleus where they transactivate target genes (Knight et al., 2012). Previous studies, have also demonstrated that the JAK-STAT pathway is also involved in the RAS pathway, mediating gene transcription (Satou and Gonzalez-Villalobos, 2012). Further, STAT3 has been shown to play a regulatory role inside mitochondria, regulating mitochondrial electron transport (Szczepanek et al., 2011; Pohjoismäki et al., 2012). In our banded hearts, as blood pressure increases due to introduction of the band, therefore, mechanisms that respond to hemodynamic overload seem to take place, including enhanced activation of RAS and increase in the cardioprotective STAT3.

Several mitochondrial protein abundances were altered in banded samples including components of mitochondrial maintenance, organization, and the electron transport system. Proteins with roles in mitochondrial fission or fusion and cell death were detected by the TMT experiment, although they were largely unchanged in banded samples compared to controls. These proteins included Dynamin-1-like protein, optic atrophy type 1, mitochondrial fragmentation-causing death-associated protein 3, and dynamin-related protein 1 (Chen et al., 2003; Chung et al., 2007). However, mitofusin 1 was slightly downregulated in banded embryos compared to controls by 1.2-fold, indicating that changes in mitochondrial fusion after banding may be partly responsible for the organizational changes observed in the 3D FIB-SEM reconstructions. Differential protein abundance of Clustered Mitochondria Homolog (CLUH) in banded samples was also detected by proteomic analysis. CLUH silencing in mammalian cells and other species alters the mitochondrial cellular distribution and leads to mitochondrial clustering in the center of the cell (Zhu et al., 1997; Fields et al., 1998; Gao et al., 2014). The clustered groups of mitochondria observed in the FIB-SEM reconstructions combined with a significant increase in CLUH protein of banded samples indicates that perhaps CLUH has an alternate role in mitochondrial biogenesis in embryonic development. The proteomic analysis also showed varied abundance of 5 large and 2 small mitochondrial ribosomal proteins (MRPs). While MRPs are not generally well-conserved among species and the roles of specific MRPs in chick cardiac development are largely unknown, the integrity of healthy mitochondrial function is thought to largely depend on normal MRP-dependent protein biosynthesis, translation, and maintenance (Sylvester et al., 2004). Proteomic response in banded samples was also detected for components of mitochondrial energy generation, including electron transport complexes, ion channels, and solute carrier proteins. Proper mitochondrial energy generation is essential for the functional health of cardiac tissue (Hüttemann et al., 2012), which may be disrupted as part of the mitochondrial reorganization in banded tissue. Other than CLUH, however, all other mitochondrial-related proteins were downregulated, suggesting a decrease in mitochondria or in mitochondrial function. Since mitochondrial cytoplasmatic volume is conserved in our studies, this downregulation is likely related to function. In fact, MAP applied to the IPA *Mitochondrial Dysfunction* canonical pathway predicts decrease of mitochondrial function and ATP. Mitochondria function depends on transcriptional regulators and nuclear-encoded mitochondrial (NEM) proteins. Since the great majority (99%) of mitochondrial proteins are encoded in the nucleus, transport of cytosolic proteins plays an important role on mitochondrial function (Boengler et al., 2011). Many NEM proteins are regulated by cAMP-responsive element binding (CREB) proteins. Interestingly, in our proteomics dataset we found a significant decrease in CREB1. The mitochondrial transcription factor A (TFAM), in addition, is a major regulator of mitochondrial DNA (mtDNA) copy number and is required for mtDNA transcription, and thus plays an important role on mitochondrial maintenance (Pohjoismäki et al., 2012). In banded heart outflow tracts, however, TFAM abundance decreased. Thus, while myofibrils and mitochondria seem more clustered in banded hearts, this could be at the expense of mitochondrial function.

The EM imaging and proteomic data taken together indicate that hemodynamic forces induced by banding after only 24 h of intervention interfere with normal development of the outflow tract myocardium. The specific molecular mechanisms by which this happens, while likely complex, remain to be elucidated and deserve additional research efforts. Interestingly, however, our data points toward the embryonic activation of the RAS pathway, known to be active in cardiac dysfunction later in life, as a possible mechanism inducing myocardial reorganization and cardiac defects in response to increase hemodynamic load. This study indicates that normal myocardial and mitochondrial reorganization is one of the initial processes affected by increased hemodynamic load, which induces a detrimental cascade of events that eventually leads to cardiac defects.

## Author contributions

MM coordinated the study, carried out a large portion of the data collection and data analysis, and drafted the manuscript; CL carried out the work done in the Multiscale Microscopy Core in the OHSU Center for Spatial Systems Biomedicine and contributed to the manuscript; LD carried out the work and data analysis done in the OHSU Proteomics Core and contributed to the manuscript; AM helped with IPA analysis, SR conceived of the study and helped draft the manuscript.

### Conflict of interest statement

The authors declare that the research was conducted in the absence of any commercial or financial relationships that could be construed as a potential conflict of interest.

## Abbreviations

CLUH: Clustered Mitochondria Homolog
FIB-SEM: Focused ion Beam Scanning Electron Microscopy
HH: Hamburger and Hamilton Development Stage
MRP: Mitochondrial Ribosomal Protein
OCT: Optical Coherence Tomography
TEM: Transmission Electron Microscopy
TMT: Tandem mass Tagging.
