# Supplementary material for: Increased Hemodynamic Load in Early Embryonic Stages Alters Myofibril and Mitochondrial Organization in the Myocardium
Source: Front Physiol. 2017 Aug 30;8:631. doi: 10.3389/fphys.2017.00631 (PMC5582297; doi:10.3389/fphys.2017.00631)
Supplement: Supplementary file 1 [file Presentation1.pptx]

## Slide 1
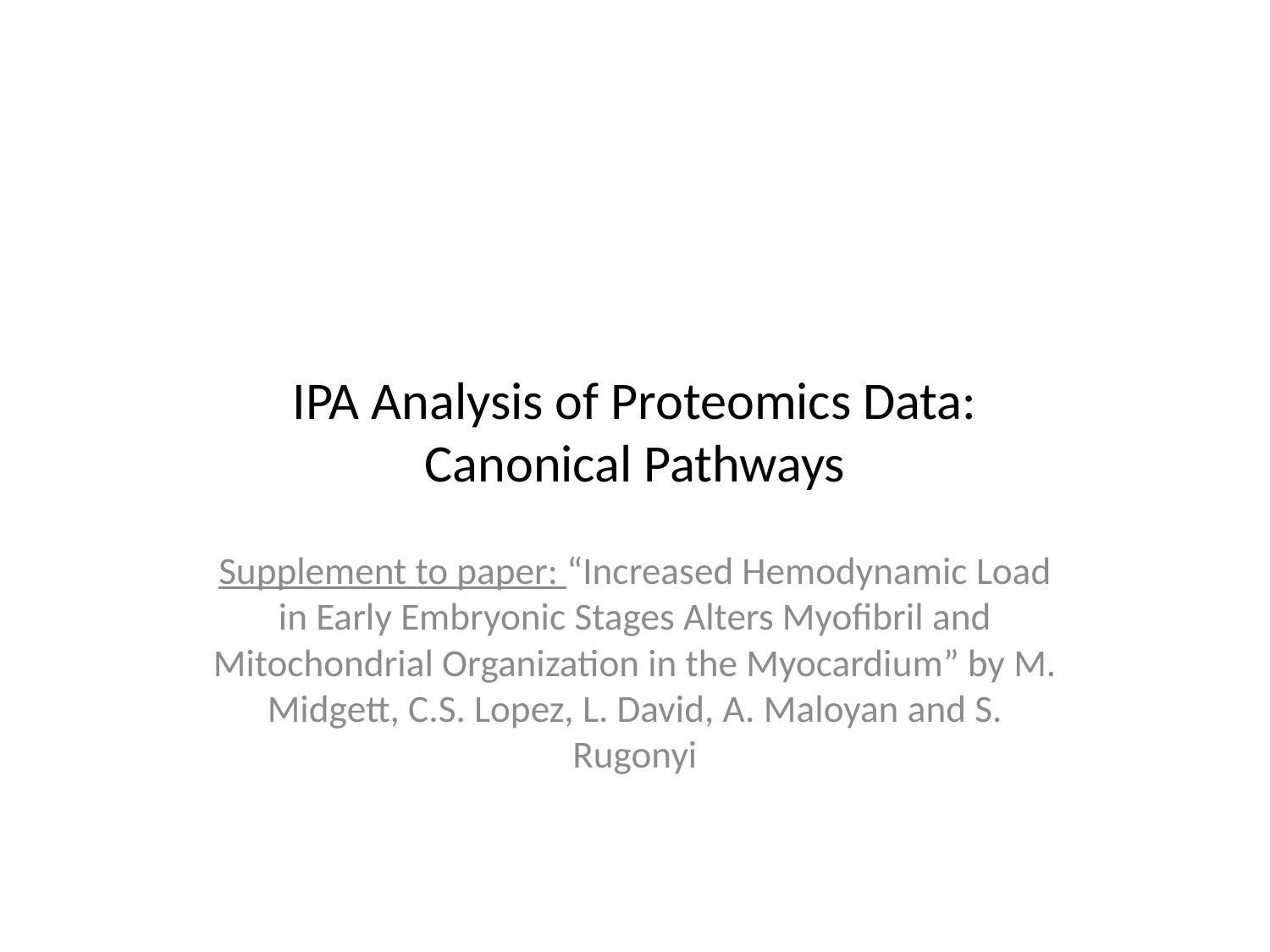

# IPA Analysis of Proteomics Data: Canonical Pathways
Supplement to paper: “Increased Hemodynamic Load in Early Embryonic Stages Alters Myofibril and Mitochondrial Organization in the Myocardium” by M. Midgett, C.S. Lopez, L. David, A. Maloyan and S. Rugonyi

## Slide 2
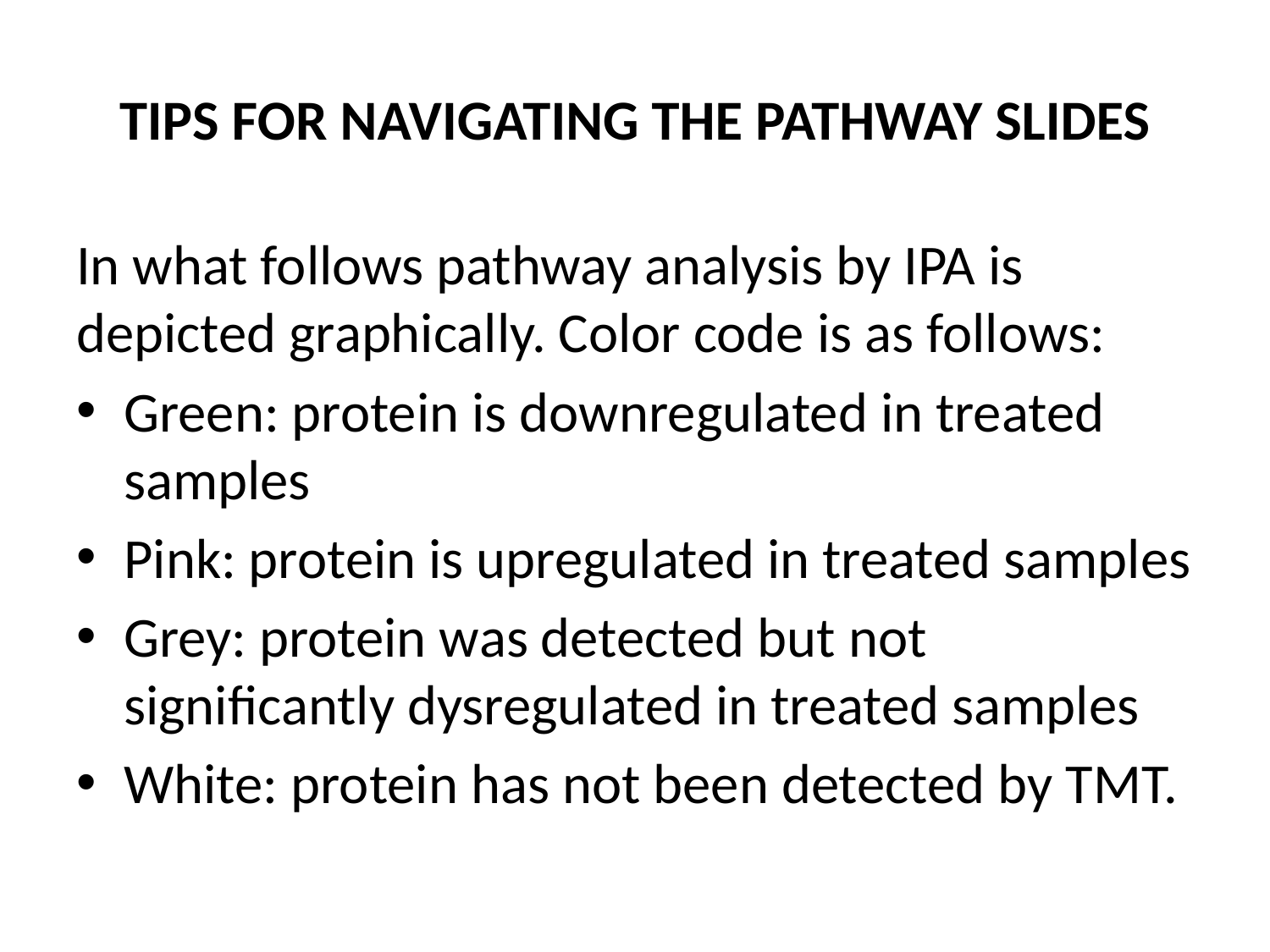

# TIPS FOR NAVIGATING THE PATHWAY SLIDES
In what follows pathway analysis by IPA is depicted graphically. Color code is as follows:
Green: protein is downregulated in treated samples
Pink: protein is upregulated in treated samples
Grey: protein was detected but not significantly dysregulated in treated samples
White: protein has not been detected by TMT.

## Slide 3
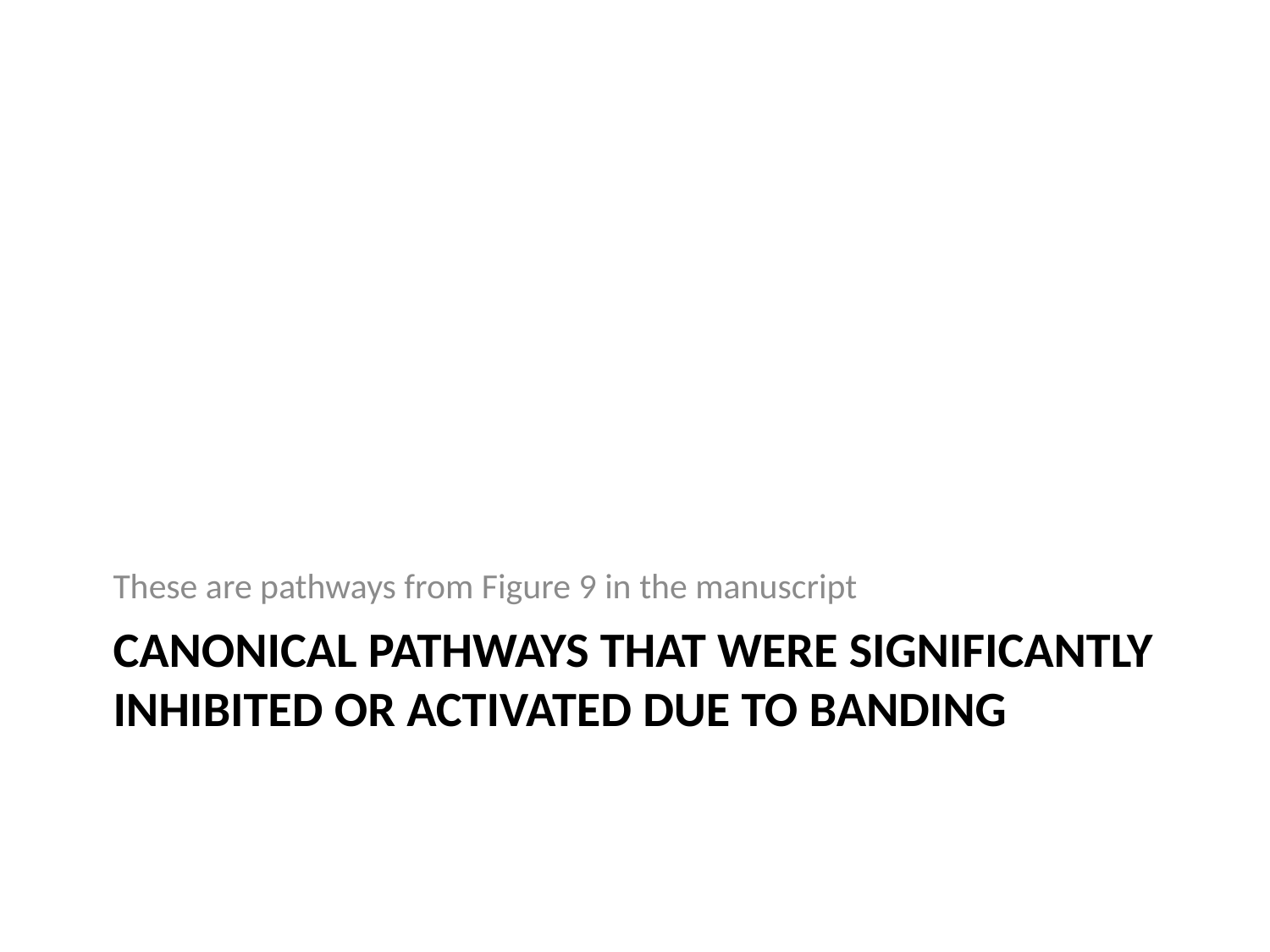

These are pathways from Figure 9 in the manuscript
# Canonical pathways that were Significantly inhibited or activated due to banding

## Slide 4
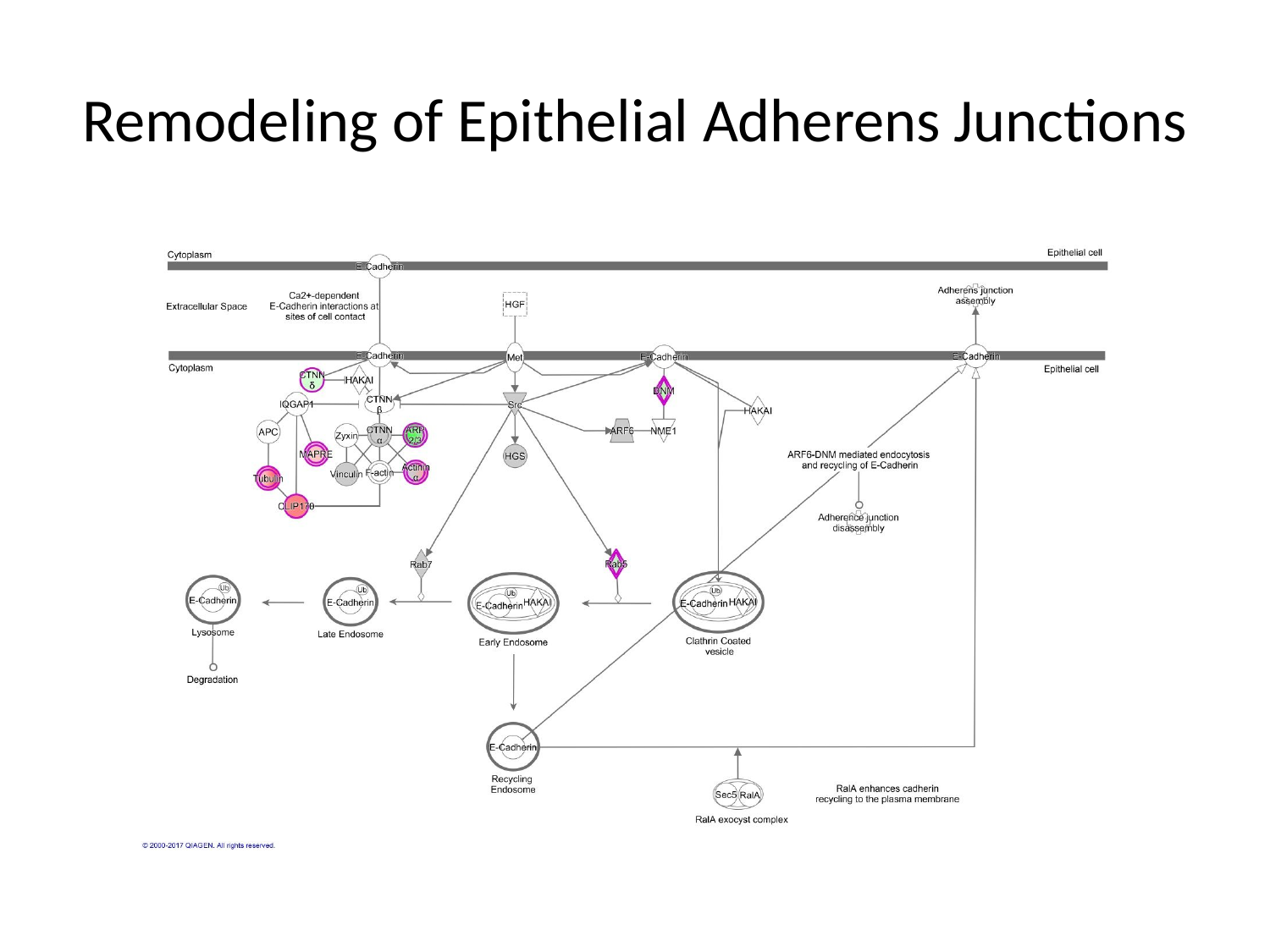

# Remodeling of Epithelial Adherens Junctions

## Slide 5
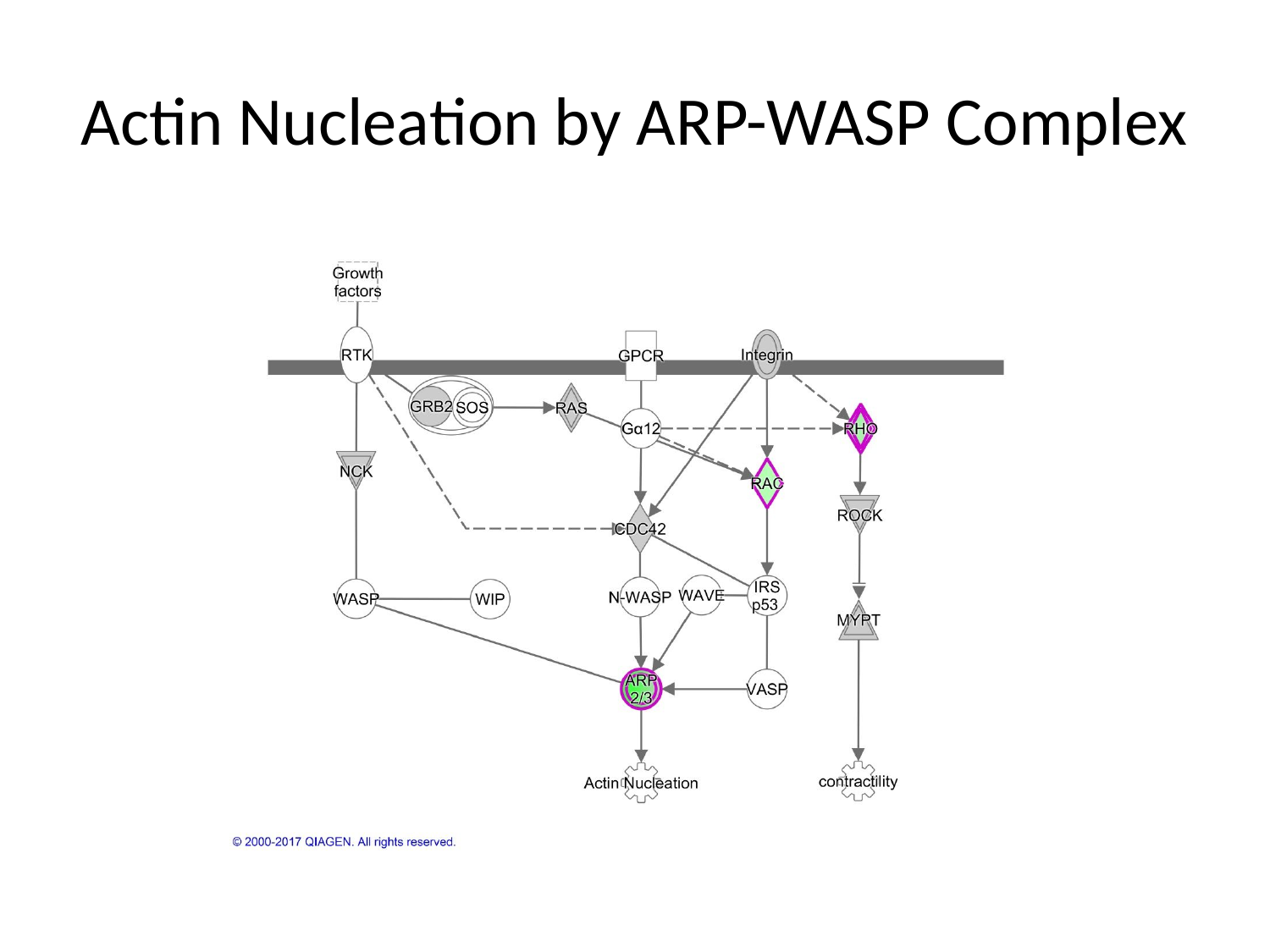

# Actin Nucleation by ARP-WASP Complex

## Slide 6
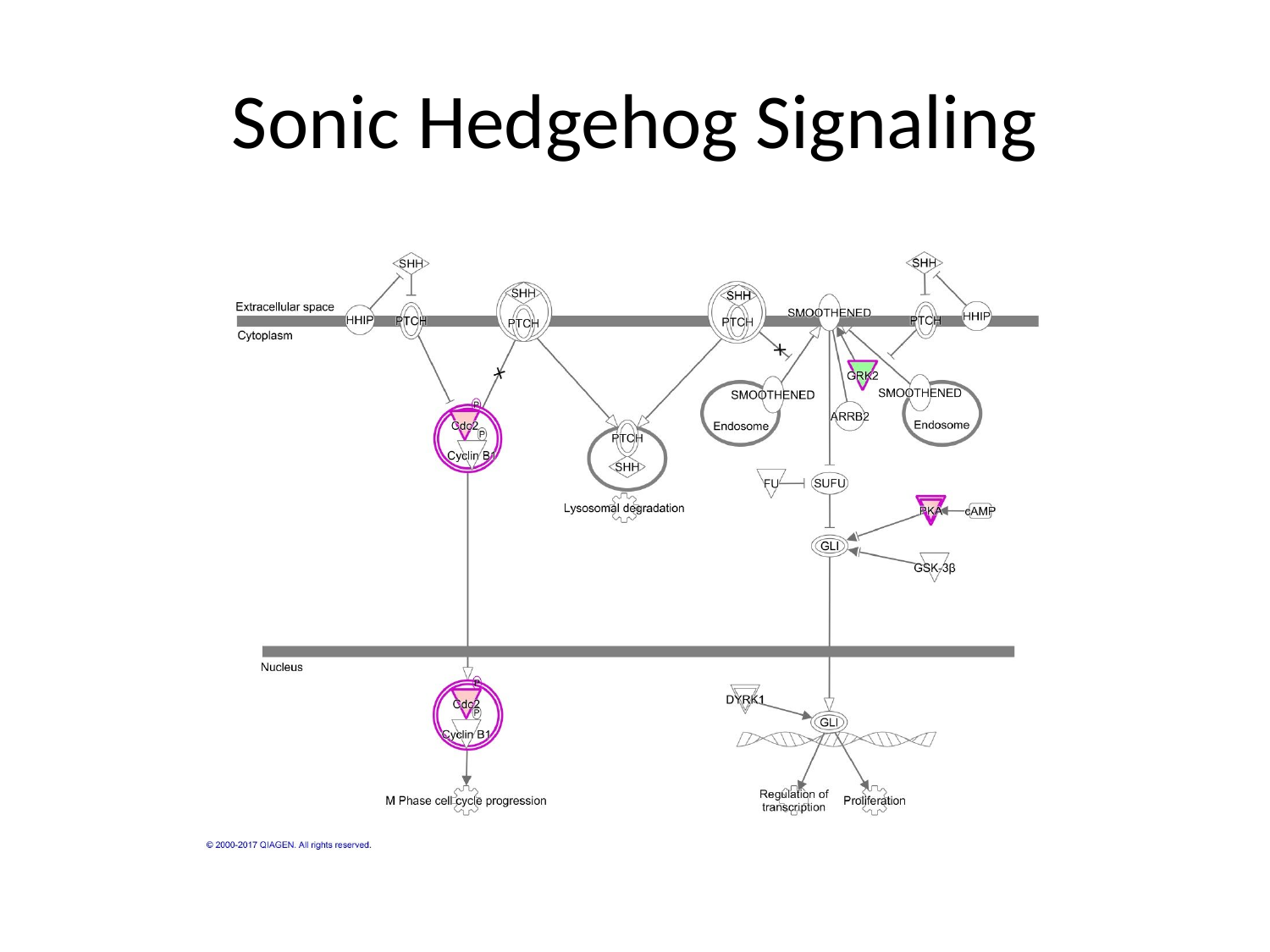

# Sonic Hedgehog Signaling

## Slide 7
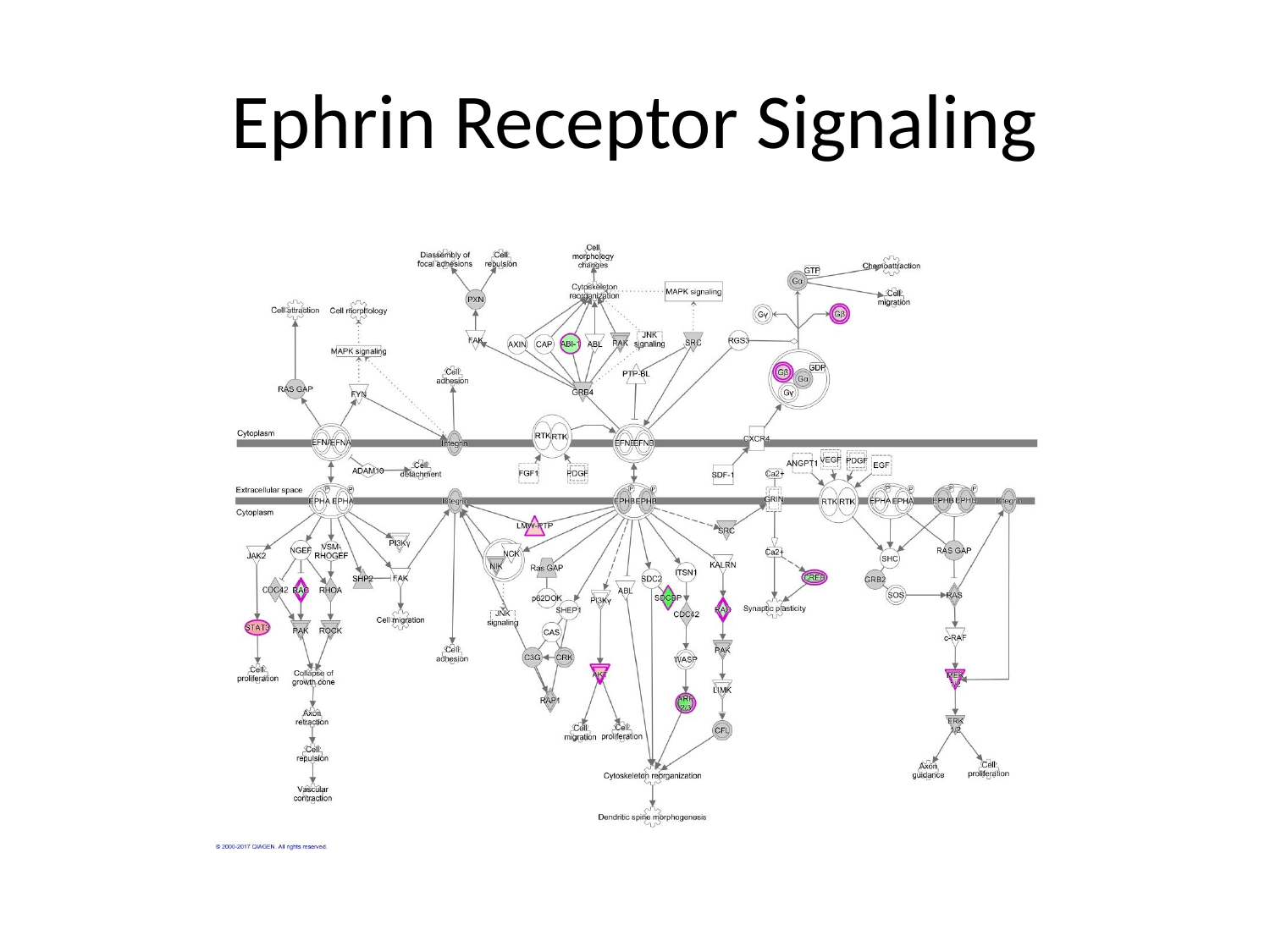

# Ephrin Receptor Signaling

## Slide 8
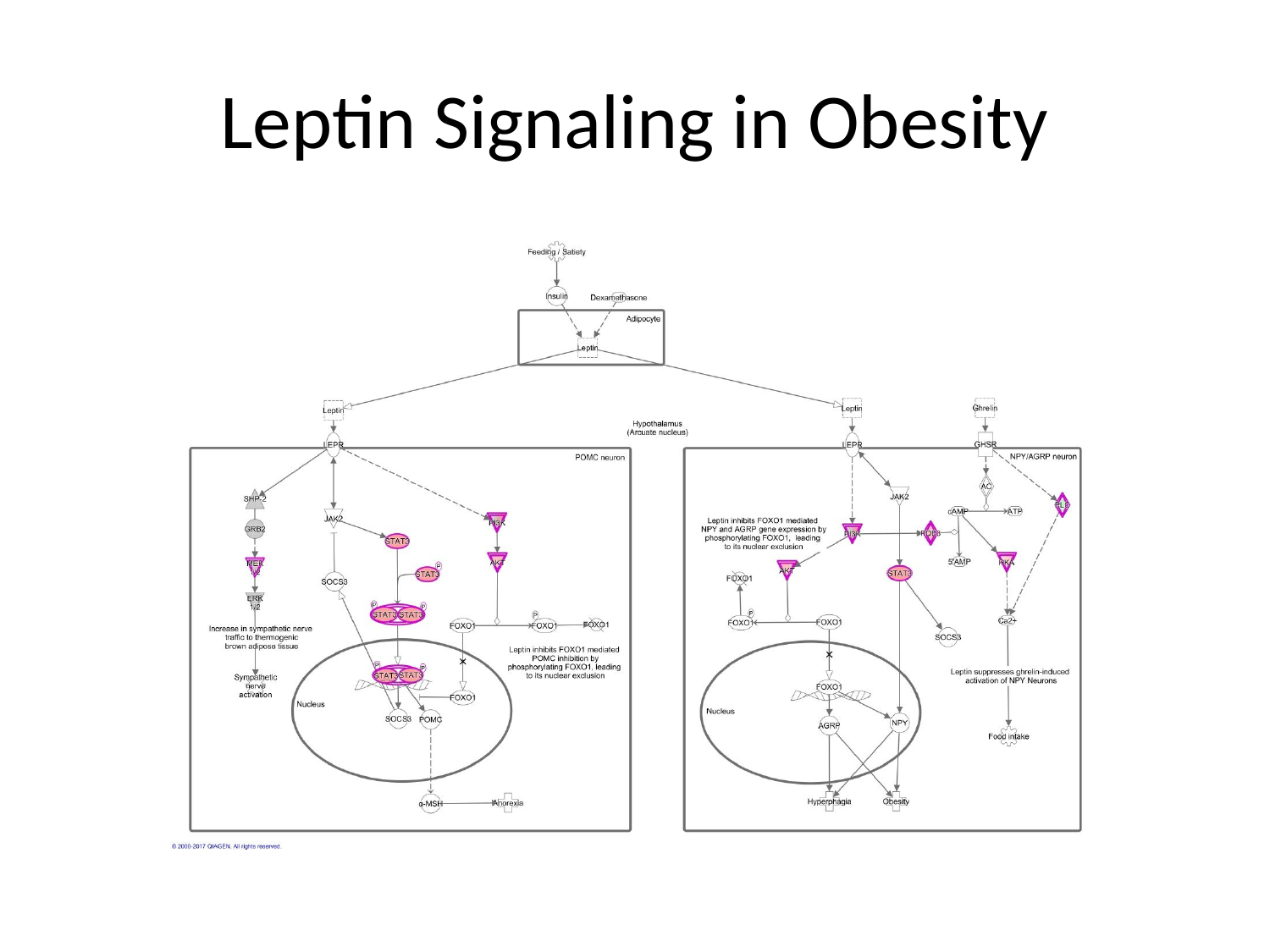

# Leptin Signaling in Obesity

## Slide 9
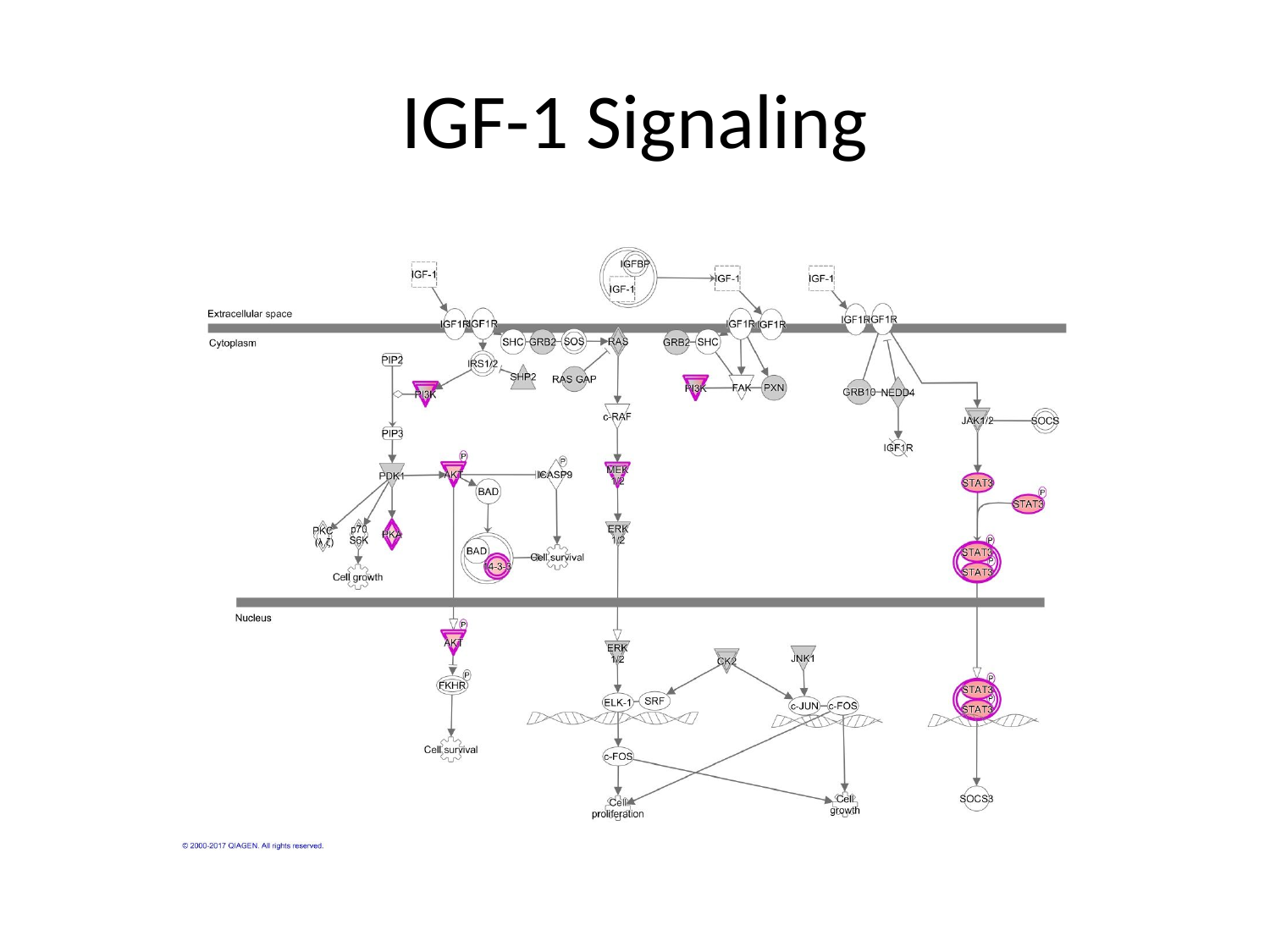

# IGF-1 Signaling

## Slide 10
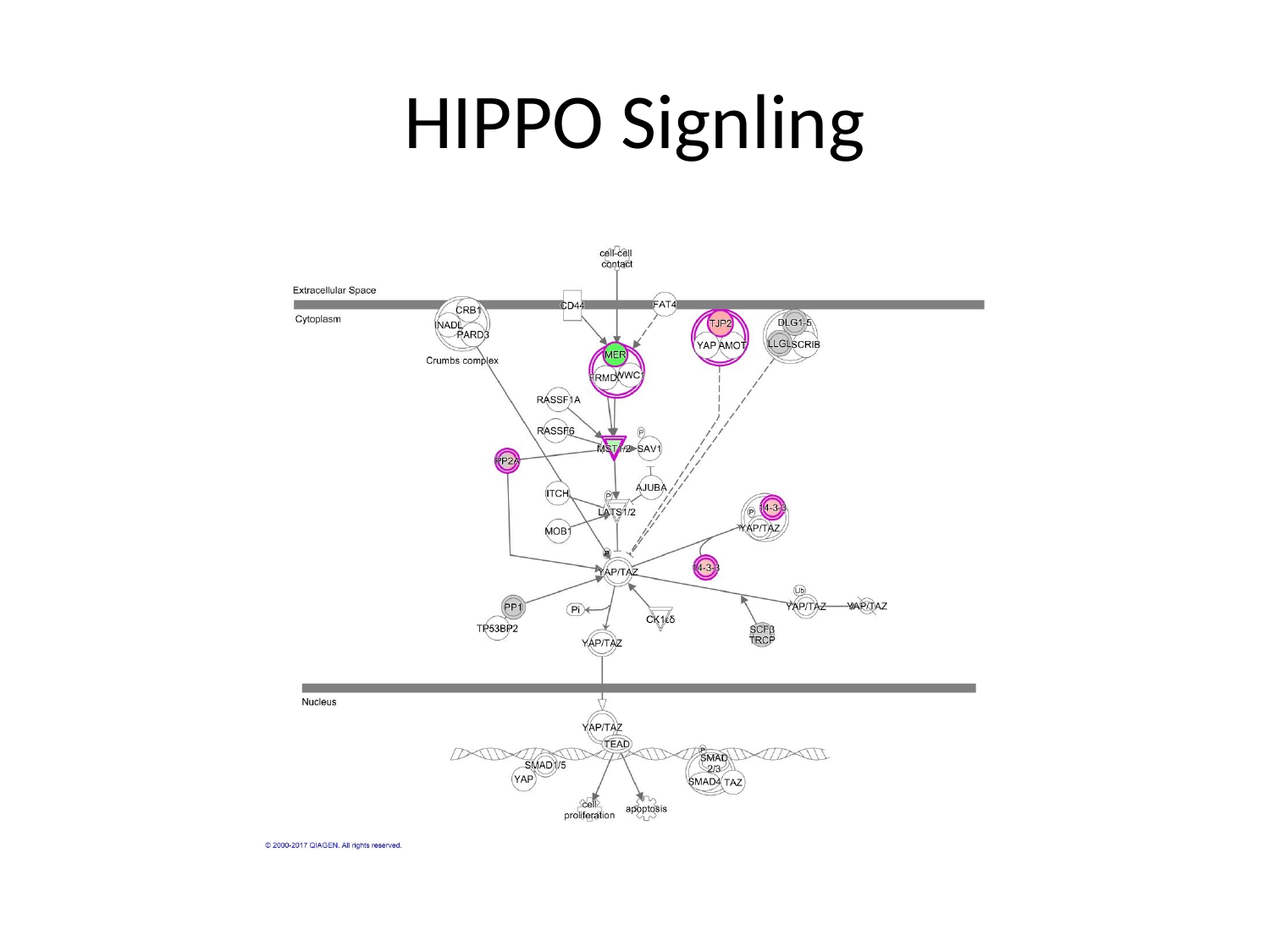

# HIPPO Signling

## Slide 11
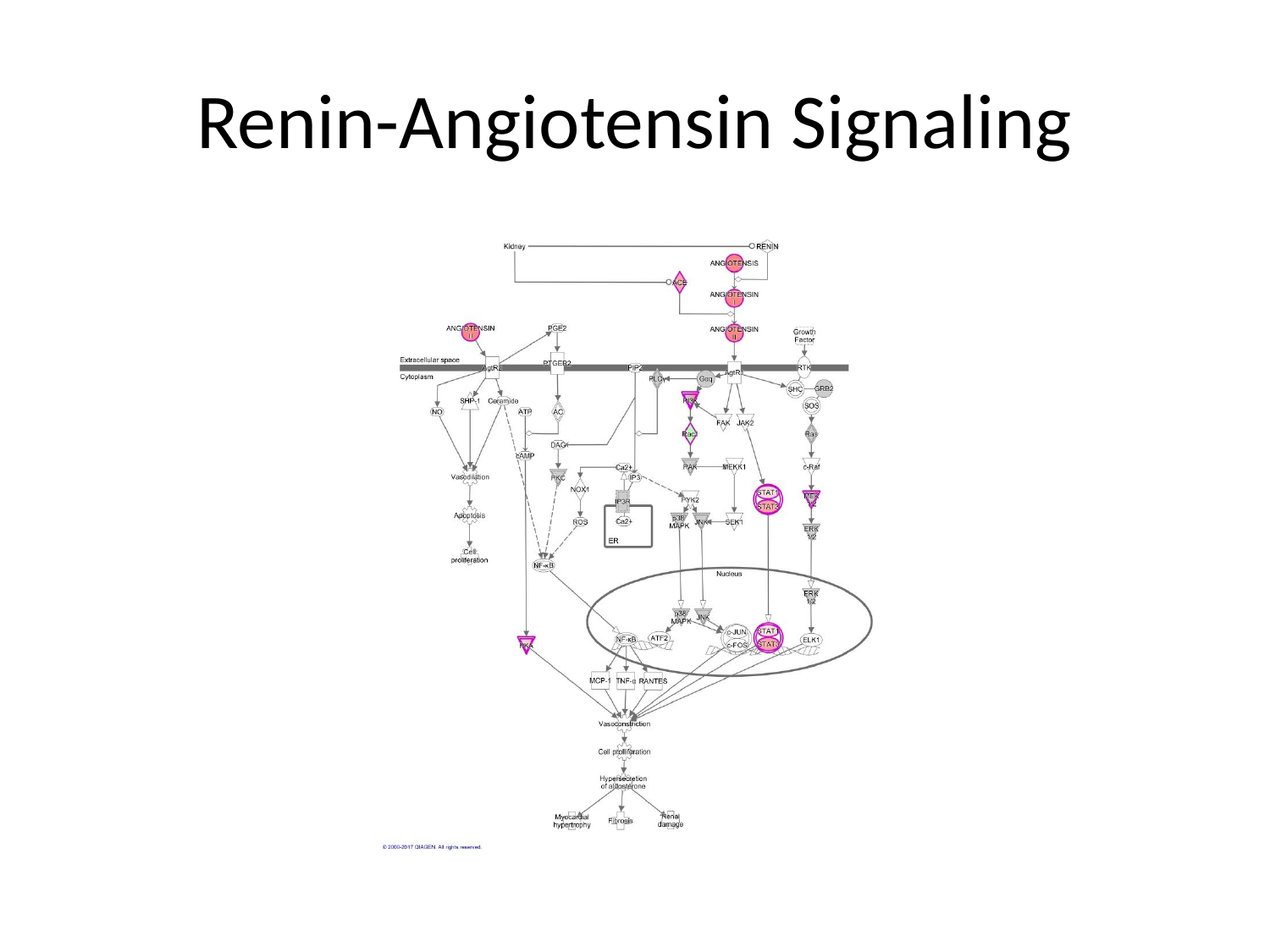

# Renin-Angiotensin Signaling

## Slide 12
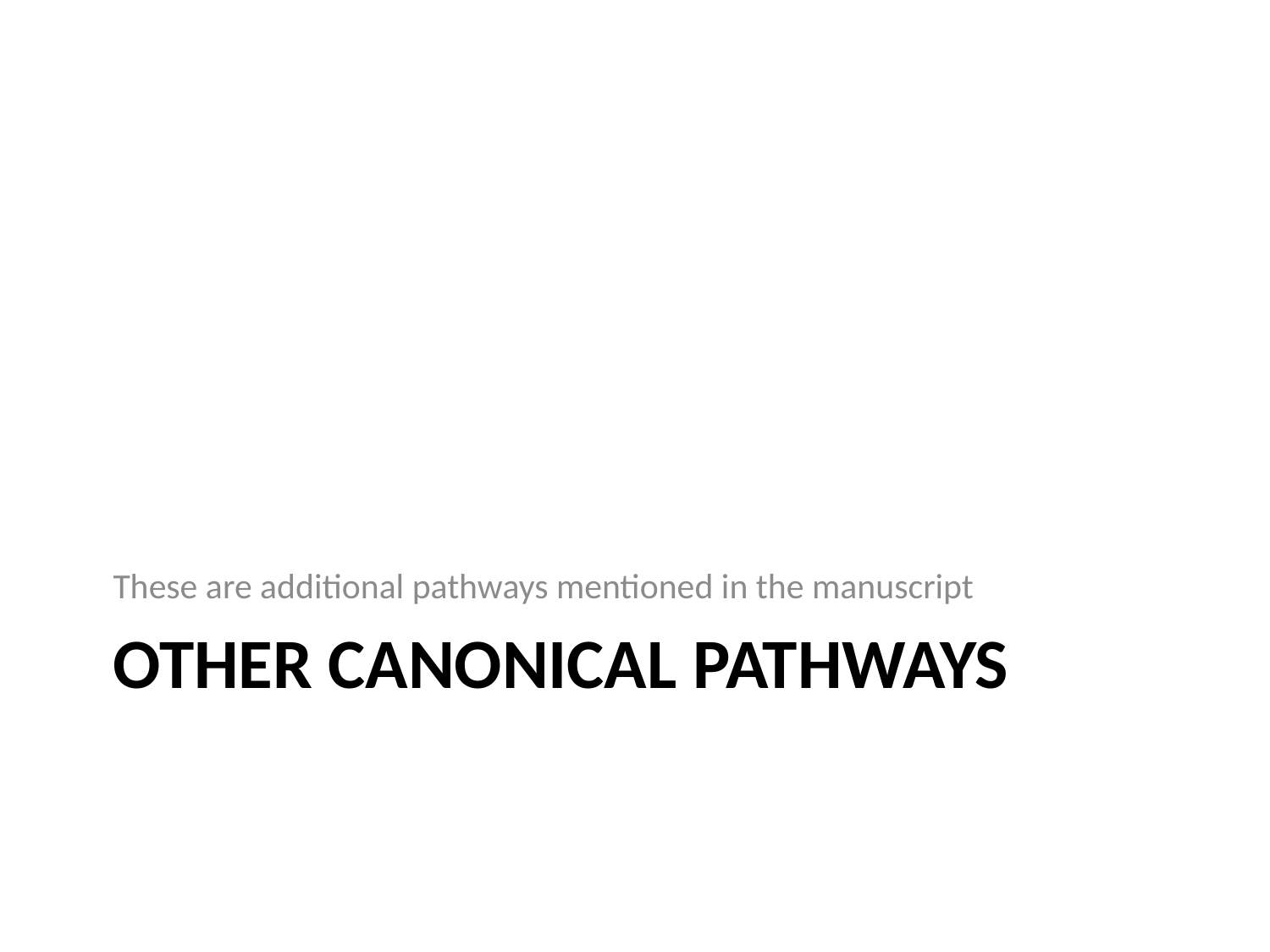

These are additional pathways mentioned in the manuscript
# Other canonical pathways

## Slide 13
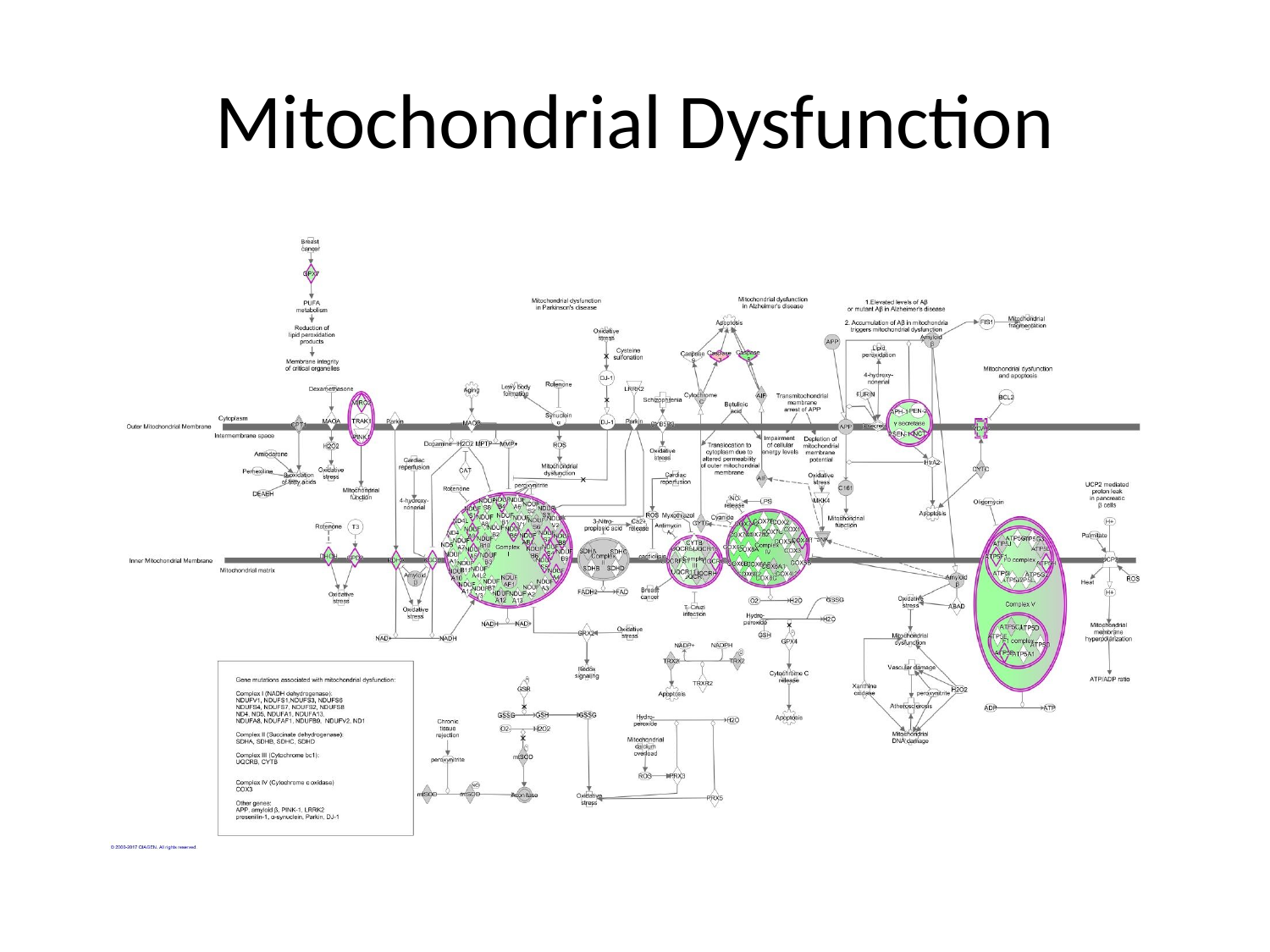

# Mitochondrial Dysfunction

## Slide 14
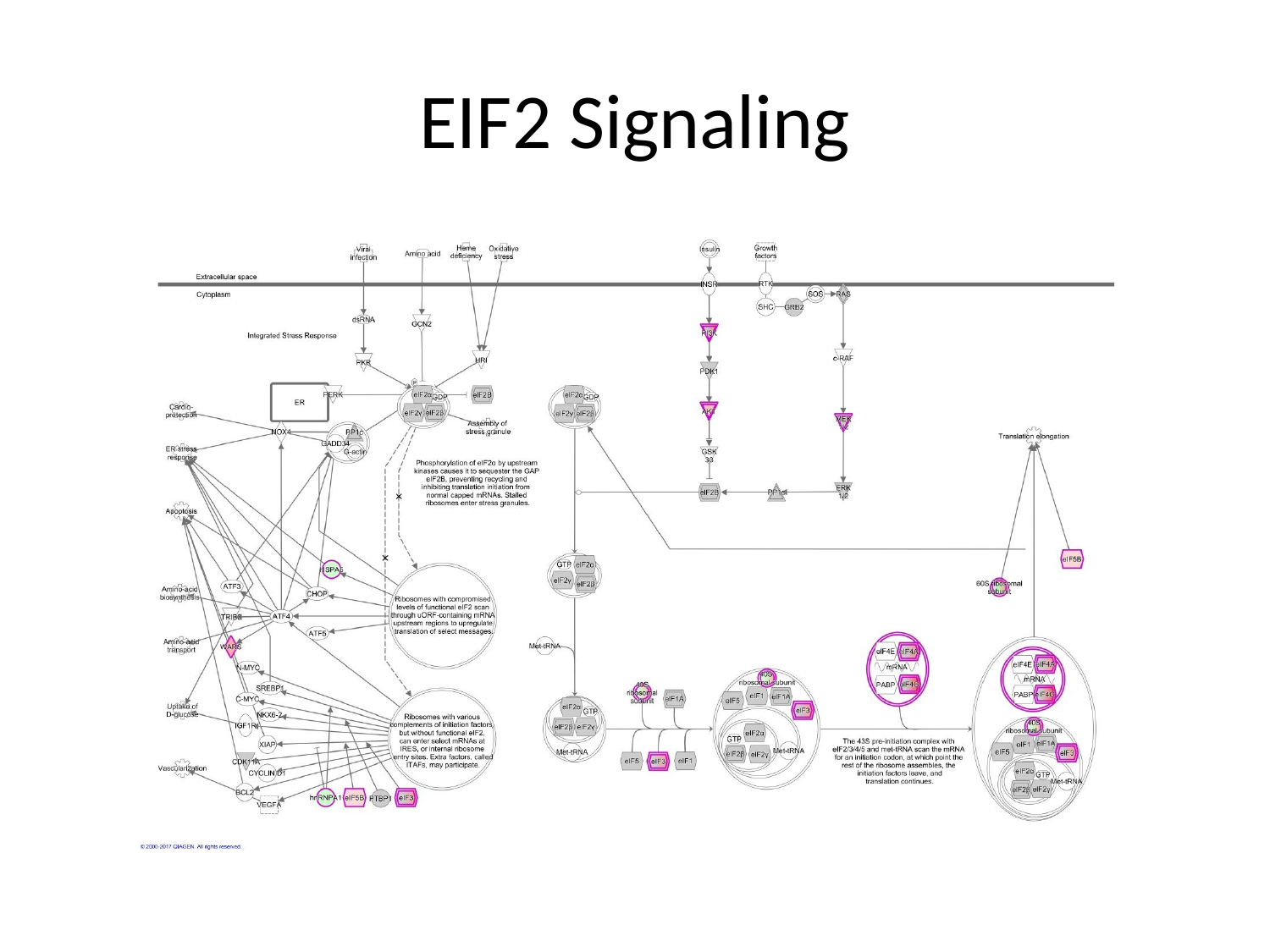

# EIF2 Signaling

## Slide 15
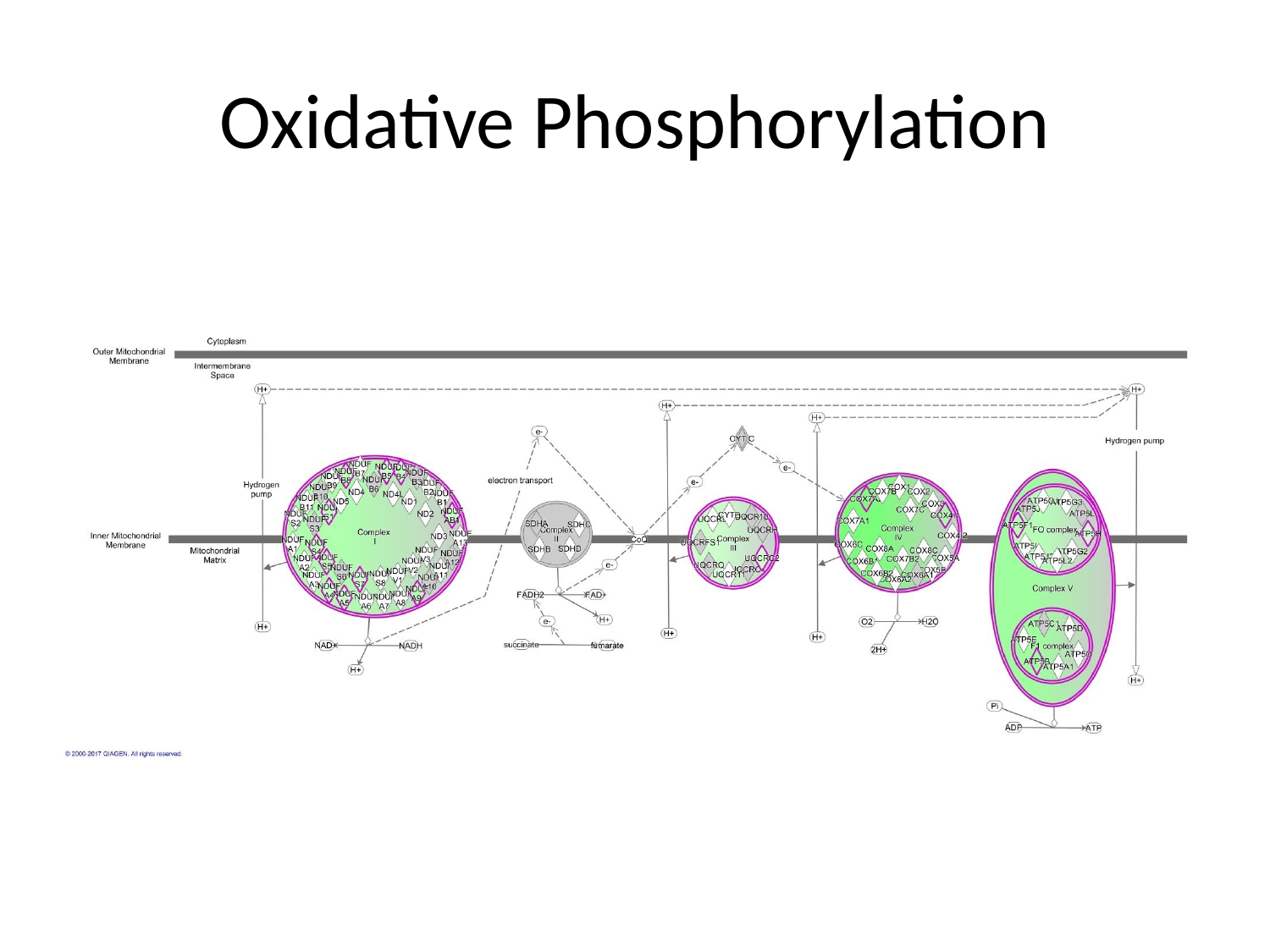

# Oxidative Phosphorylation

## Slide 16
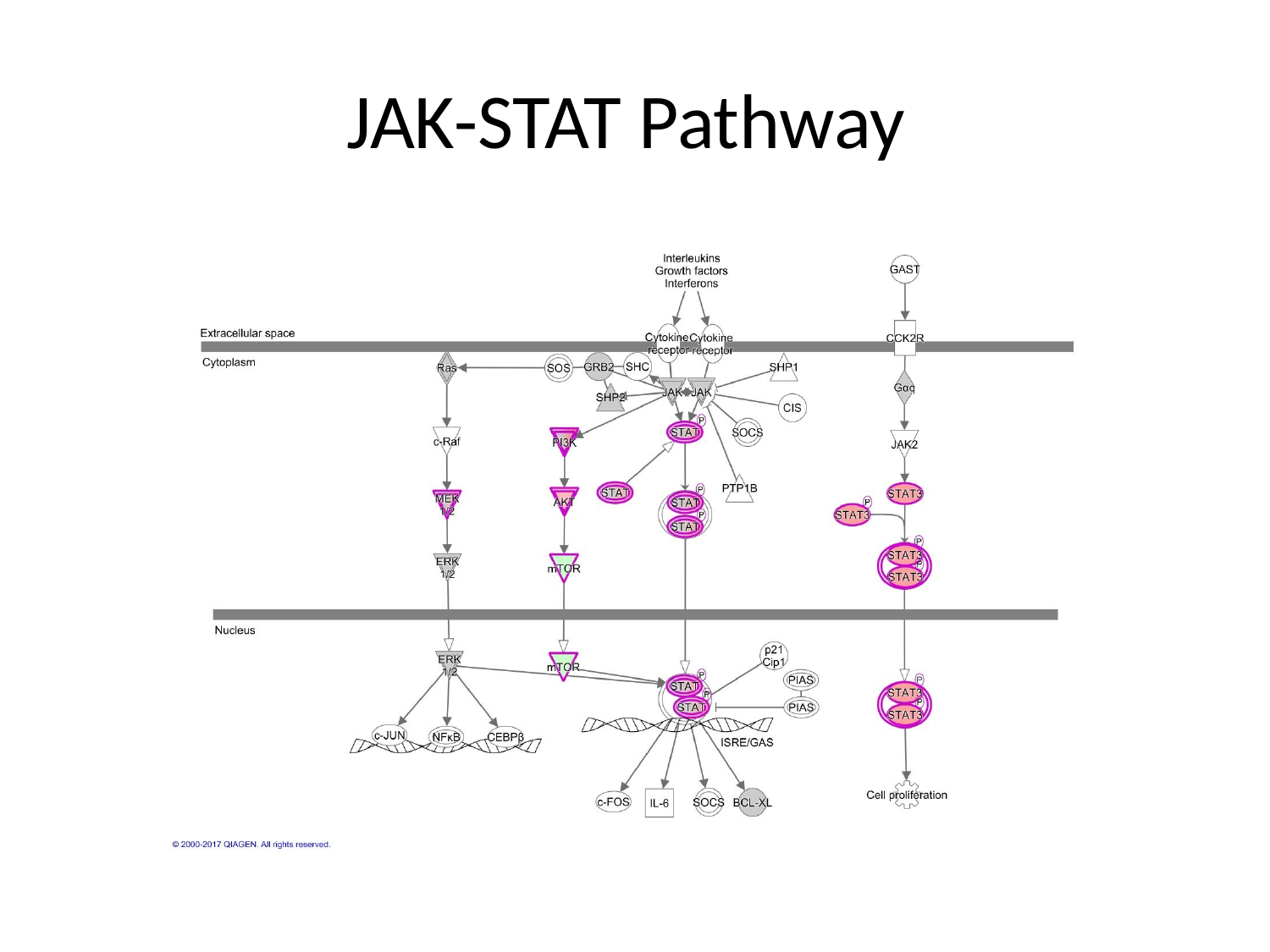

# JAK-STAT Pathway

## Slide 17
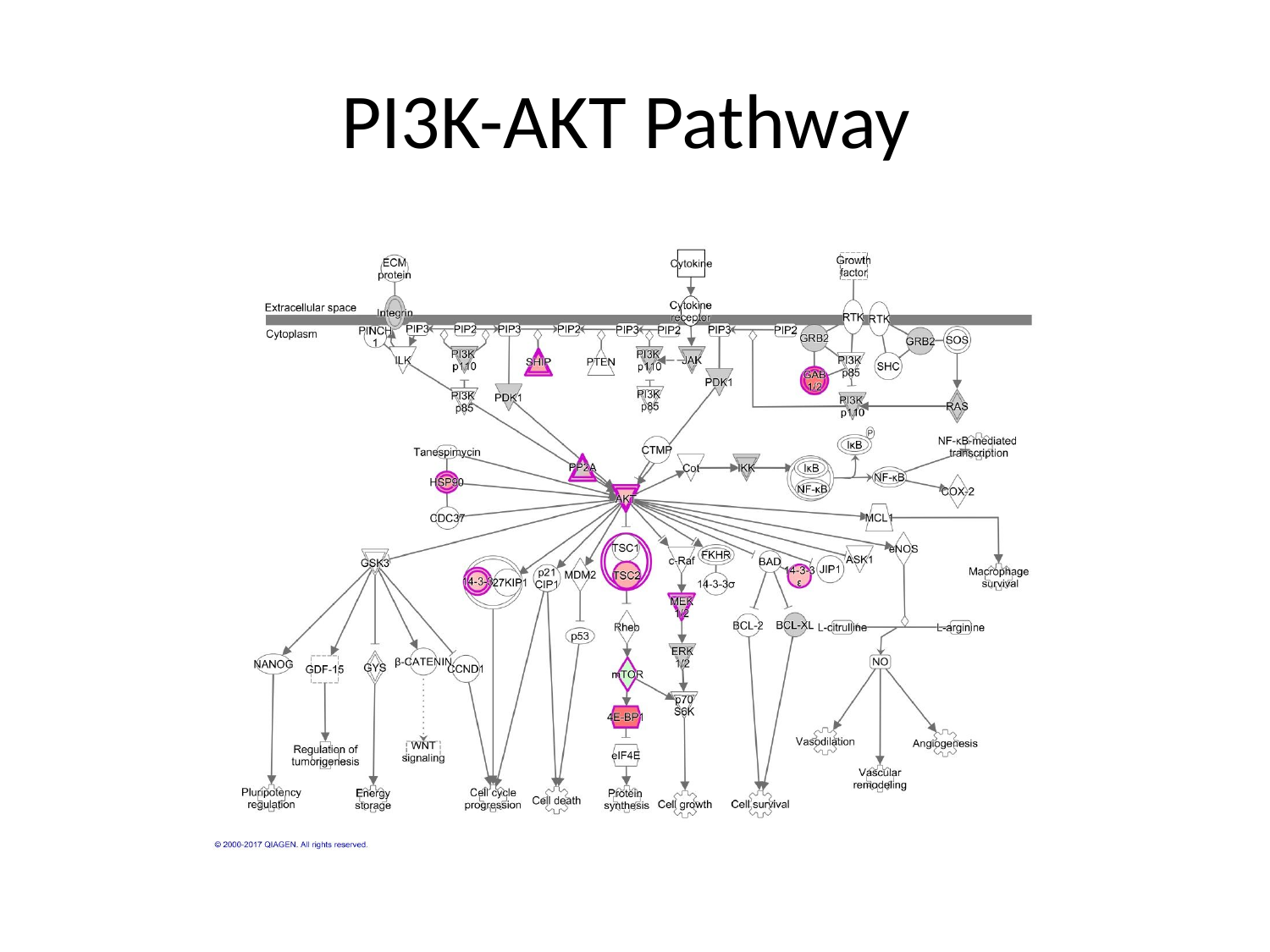

# PI3K-AKT Pathway
